# Supplementary material for: Motif-centric phosphoproteomics to target kinase-mediated signaling pathways
Source: Cell Rep Methods. 2022 Jan 14;2(1):100138. doi: 10.1016/j.crmeth.2021.100138 (PMC9017188; doi:10.1016/j.crmeth.2021.100138)
Supplement: Document S1. Figures S1–S4 and Tables S1–S3 [file mmc1.pdf]

Cell Reports Methods, Volume *2*

Supplemental information

Motif-centric phosphoproteomics to target kinase-mediated signaling pathways

Chia-Feng Tsai, Kosuke Ogata, Naoyuki Sugiyama, and Yasushi Ishihama

Supplemental Information

Motif-centric phosphoproteomics to target kinase-mediated signaling pathways

Chia-Feng Tsai[1,3,4], Kosuke Ogata[1,3], Naoyuki Sugiyama[1], Yasushi Ishihama[1,2]

[1]Graduate School of Pharmaceutical Sciences, Kyoto University, Kyoto 606-8501, Japan.

[2]Laboratory of Clinical and Analytical Chemistry, National Institute of Biomedical Innovation, Health and Nutrition, Ibaraki, Osaka, 567-0085, Japan.

[3]These authors contributed equally.

[4]Current address: Biological Sciences Division, Pacific Northwest National Laboratory, Richland, WA 99354, USA.

*Correspondence and lead contact: yishiham@pharm.kyoto-u.ac.jp

Tel: +81-75-753-4555

Fax: +81-75-753-4601

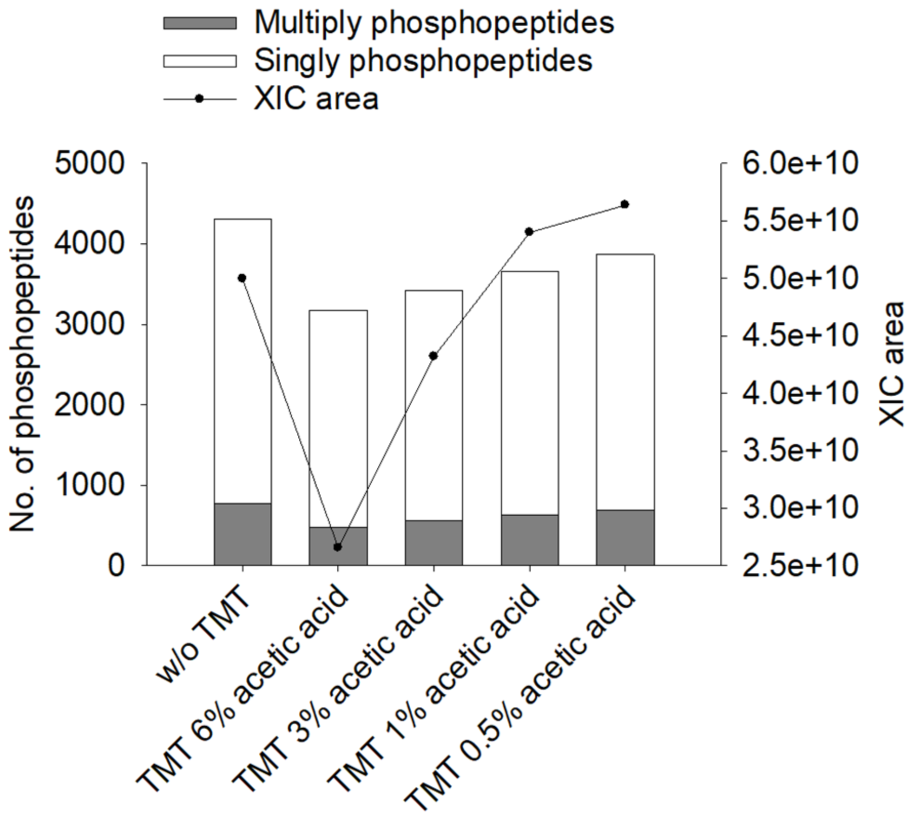

Figure S1. Effects of acetic acid concentration in the loading buffer on the enrichment of TMT-phosphopeptides by IMAC. TMT0 reagent was used for labeling 50 μg of HeLa tryptic peptides and IMAC enrichment followed by LC/MS/MS measurement with label free quantitation was performed for each sample (n=1). The optimized condition was used for phosphopeptide enrichment shown in Figure 1a.

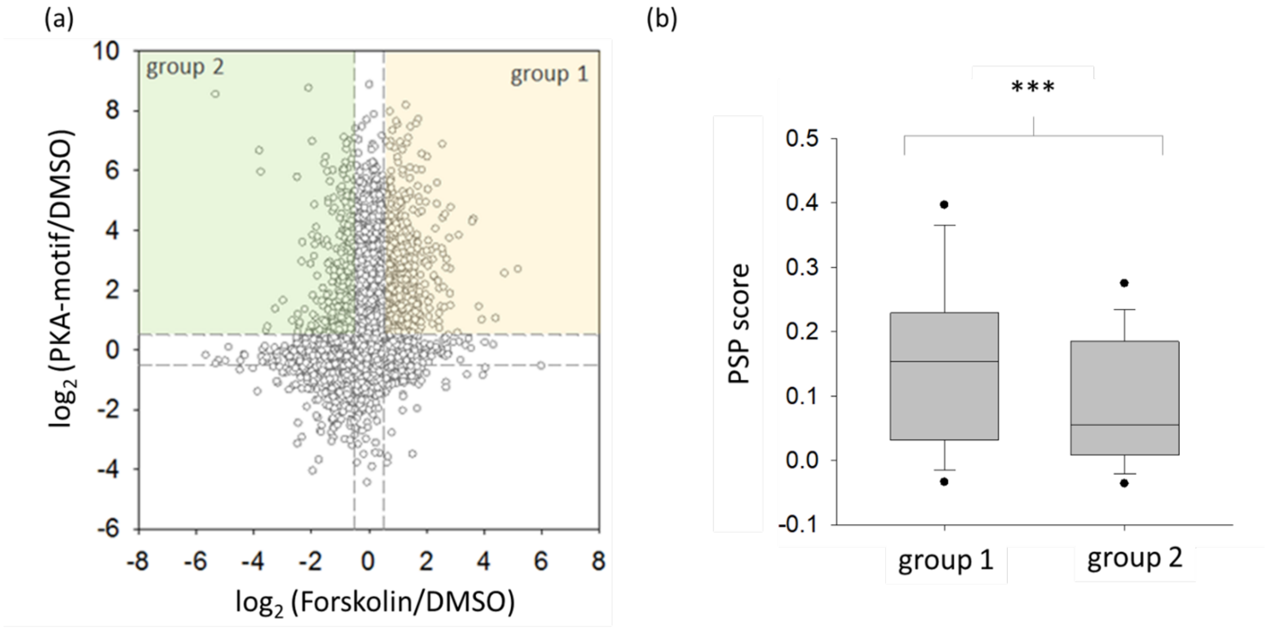

Figure S2. PKA motif-centric phosphoproteome analysis. Related to acidophilic CK2 phosphorylation shown in Figure 2, but *in vitro* phosphorylation was done at protein level in this case. (a) Signal intensity ratios of 620 phosphorylation sites phosphorylated *in vitro* by PKA increased after forskolin treatment (group 1), while 322 phosphorylation sites phosphorylated *in vitro* by PKA were inhibited by forskolin (group 2). (b) PSP score distribution of group 1 and group 2. *** $p<0.001$. Duplicate sample preparation and triplicate LC/MS/MS analyses were performed for each TMT set as shown in Table S3.

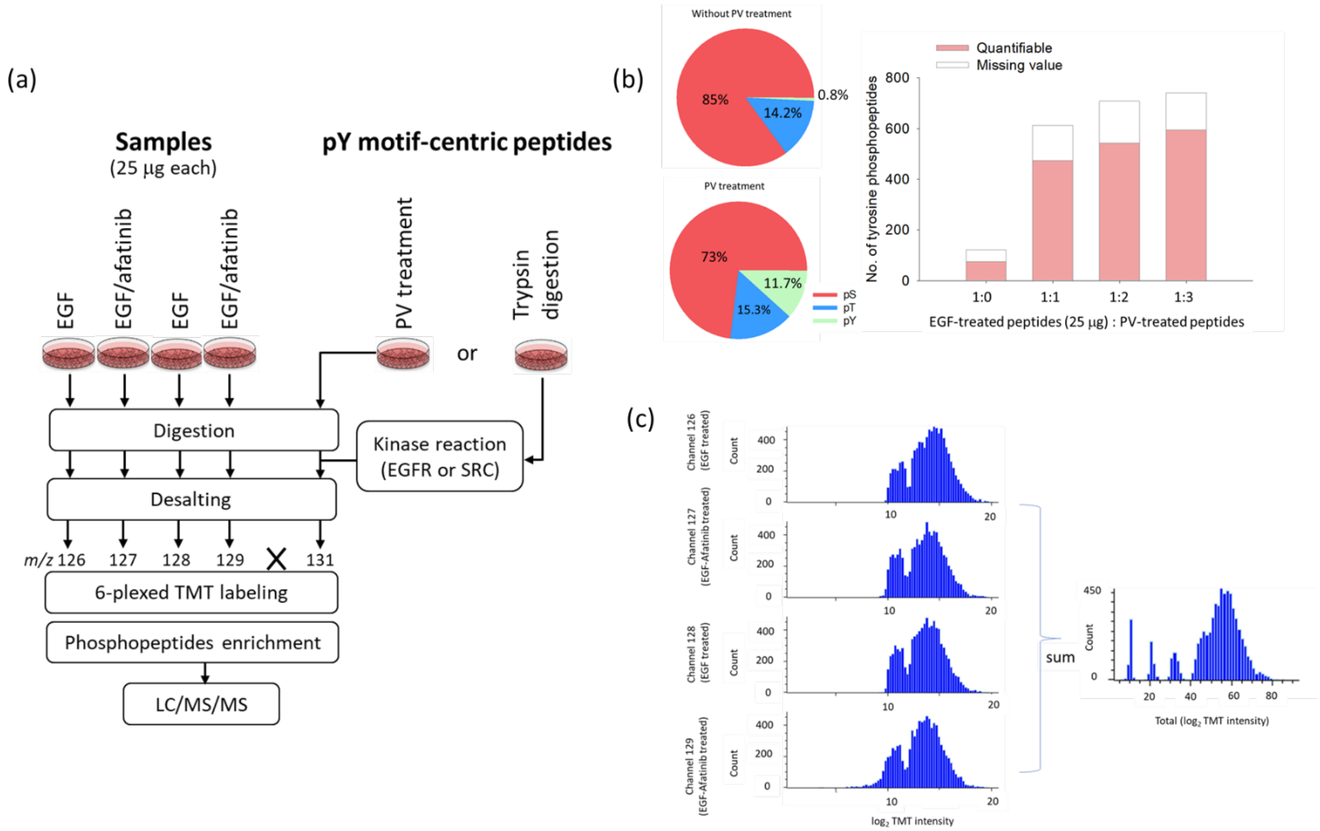

Figure S3. Experimental design for isobaric pY motif-centric phosphoproteomic approach. Related to Figure 3. (a) The pY-centric samples were generated by PV treatment or *in vitro* kinase reaction with EGFR or SRC. The tryptic peptides from study samples and pY motif-centric samples were labeled with multiplexed TMT tags, and were enriched by IMAC and analyzed by LC/MS/MS. (b) Effects of PV treatment on pY identification**.** Contents of pS, pT and pY sites identified from the IMAC-enriched tryptic peptides from PV-treated HeLa cells and untreated HeLa cells are shown in the pie charts (left). The numbers of quantifiable pY peptides from 25 µg of EGF-treated HeLa peptides, 25 µg of EGF/afatinib-treated HeLa peptides and different amounts of PV-treated peptides analyzed according to the workflow (a) are shown (right). A quantifiable pY peptide is defined as a pY peptide that contains the signals of TMT reporter ions in all TMT channels of the MS^3 spectrum. Otherwise, pY peptides are considered as missing values. (c) TMT signal distribution in each TMT reporter channel (left) and the merged signals from all channels except the motif-centric channel (right). Duplicate sample preparation for each TMT set was performed as shown in Table S3.

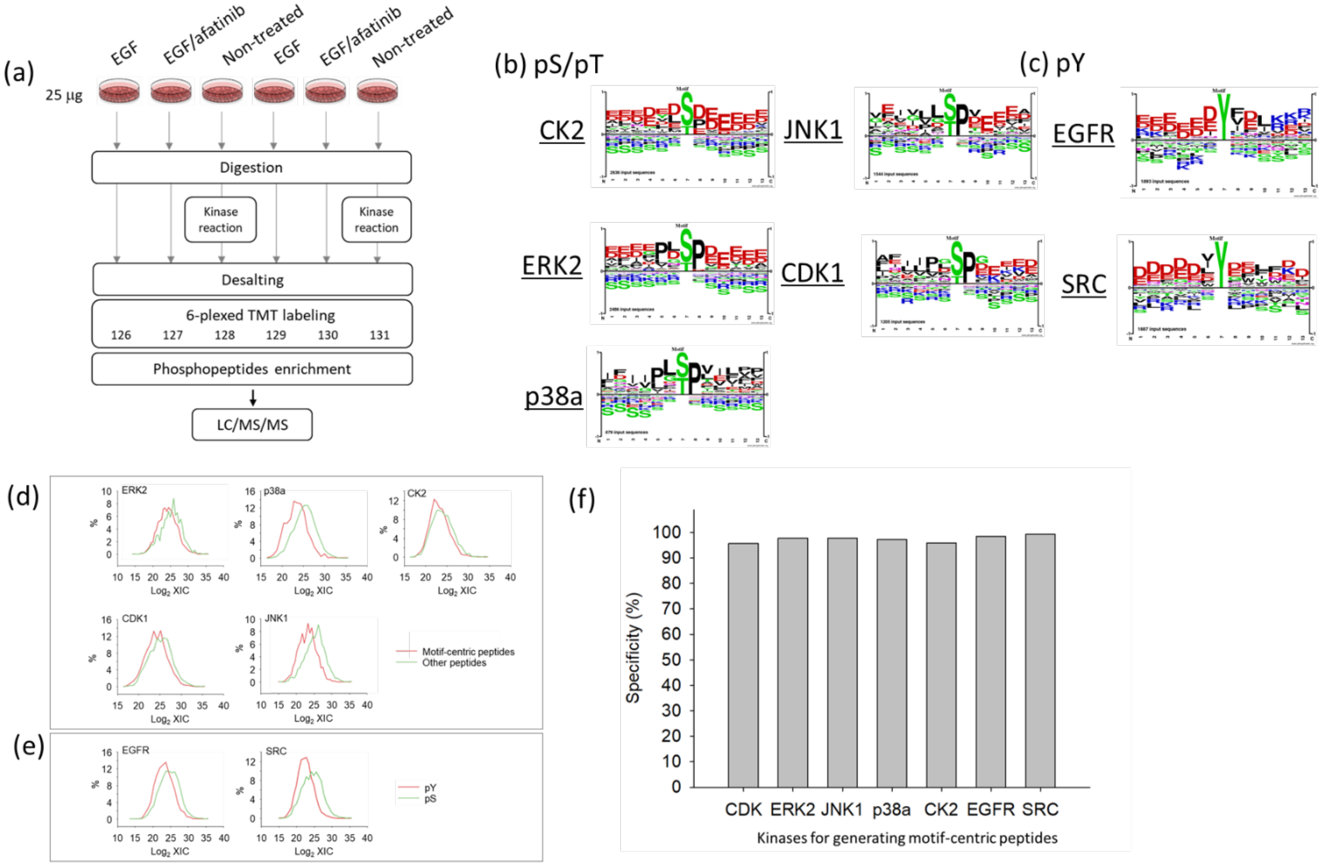

Figure S4. Isobaric motif-centric phosphoproteomics results with multiple kinases. Related to Figure 5. (a) Workflow of isobaric motif-centric phosphoproteomic approach with multiple kinases. Seven kinases, i.e., Pro-directed kinases ((ERK1 (MAPK3), JNK1(MAPK8), P38a (MAPK14) and CDK1)), tyrosine kinases (SRC and EGFR) and acidophilic kinase (CK2), were employed to generate motif-centric phosphopeptides. The tryptic peptides from study samples and motif-centric peptides were labeled with 6-plexed TMT tags. The TMT labeling phosphopeptides were enriched by IMAC and analyzed by LC/MS/MS. (b) Phosphorylation motif analysis for the motif-centric pS/pT sites. (c) Phosphorylation motif analysis for the motif-centric pY sites. (d) The distribution of XIC signals in MS1 for endogenous pS/pT peptides. (e) The distribution of XIC signals in MS1 for endogenous pY peptides. The XIC signals of the endogenous phosphopeptides in MS1 were calculated based on the TMT ratio between study samples and the motif-centric sample. (f) IMAC enrichment specificity for phosphopeptides based on peptide counts for each kinase experiment. Duplicate sample preparation and 2 or 3 replicate LC/MS/MS analyses were performed for each kinase as shown in Table S3.

Table S1. GO enrichment analysis of downregulated CK2-motif centric peptides (related to Figure 2d).

#Term ID	Term description	Observed gene count	Background gene count	False discovery rate
**Molecular function**				
GO:0005488	binding	168	11878	1.21E-11
GO:0005515	protein binding	112	6605	7.07E-09
GO:0005524	ATP binding	44	1462	7.07E-09
GO:0097159	organic cyclic compound binding	98	5382	7.07E-09
GO:1901363	heterocyclic compound binding	97	5305	7.07E-09
GO:0000166	nucleotide binding	53	2097	1.74E-08
GO:0016887	ATPase activity	22	392	1.74E-08
GO:0035639	purine ribonucleoside triphosphate binding	48	1794	1.78E-08
GO:0032555	purine ribonucleotide binding	48	1853	4.70E-08
GO:0008144	drug binding	45	1710	9.06E-08
GO:0036094	small molecule binding	56	2460	1.01E-07
GO:0043168	anion binding	59	2696	1.31E-07
GO:0017111	nucleoside-triphosphatase activity	28	778	2.68E-07
GO:0003676	nucleic acid binding	66	3332	5.02E-07
GO:0097367	carbohydrate derivative binding	50	2163	5.02E-07
GO:0051082	unfolded protein binding	10	106	6.28E-06
GO:0003677	DNA binding	51	2457	7.84E-06
GO:0043167	ion binding	92	6066	5.98E-05
GO:0042623	ATPase activity, coupled	14	320	0.00015
GO:0004386	helicase activity	9	147	0.00059
GO:0070182	DNA polymerase binding	4	15	0.0007
GO:0008026	ATP-dependent helicase activity	7	90	0.001
GO:0044877	protein-containing complex binding	24	968	0.001
GO:0019899	enzyme binding	41	2197	0.0014
GO:0004004	ATP-dependent RNA helicase activity	6	66	0.0015
GO:0140097	catalytic activity, acting on DNA	9	173	0.0016
GO:0008092	cytoskeletal protein binding	21	882	0.0043
GO:0008017	microtubule binding	10	253	0.005
GO:0016787	hydrolase activity	42	2448	0.0056
GO:0003824	catalytic activity	78	5592	0.0079
GO:0003697	single-stranded DNA binding	6	99	0.0094
GO:0008094	DNA-dependent ATPase activity	5	66	0.0102
GO:0015631	tubulin binding	11	344	0.0124
GO:0003918	DNA topoisomerase type II (ATP-hydrolyzing) activity	2	3	0.0134
GO:0003723	RNA binding	19	850	0.0142
GO:0042826	histone deacetylase binding	6	110	0.0142
GO:0019903	protein phosphatase binding	6	123	0.0234
GO:0004849	uridine kinase activity	2	5	0.025
GO:0016879	ligase activity, forming carbon-nitrogen bonds	4	50	0.025
GO:0008327	methyl-CpG binding	3	25	0.0317
GO:0044183	protein folding chaperone	3	25	0.0317
GO:0042802	identical protein binding	30	1754	0.034
GO:0008494	translation activator activity	2	7	0.0384
GO:0003729	mRNA binding	7	198	0.049
**Cellular component**				
GO:0044428	nuclear part	117	4359	2.28E-26
GO:0031981	nuclear lumen	109	4030	3.48E-24
GO:0070013	intracellular organelle lumen	123	5162	5.11E-24
GO:0005654	nucleoplasm	97	3446	4.01E-22
GO:0005634	nucleus	138	6892	1.69E-21

GO:0043226	organelle	181	12432	6.80E-19
GO:0044422	organelle part	155	9111	8.73E-19
GO:0044446	intracellular organelle part	152	8882	2.97E-18
GO:0044424	intracellular part	189	13996	6.57E-18
GO:0043229	intracellular organelle	177	12193	3.35E-17
GO:0005829	cytosol	107	4958	1.75E-16
GO:0043232	intracellular non-membrane-bounded organelle	94	4005	4.54E-16
GO:0043231	intracellular membrane-bounded organelle	160	10365	1.83E-15
GO:0043227	membrane-bounded organelle	164	11244	1.37E-13
GO:0032991	protein-containing complex	97	4792	1.43E-12
GO:1990904	ribonucleoprotein complex	35	770	3.53E-12
GO:0044464	cell part	193	16244	4.77E-11
GO:0005730	nucleolus	33	926	8.32E-09
GO:0005737	cytoplasm	149	11238	1.05E-06
GO:0044444	cytoplasmic part	131	9377	1.69E-06
GO:0030684	preribosome	9	74	2.66E-06
GO:0005694	chromosome	28	950	8.16E-06
GO:0030687	preribosome, large subunit precursor	6	24	8.16E-06
GO:0016607	nuclear speck	17	381	8.88E-06
GO:0044452	nucleolar part	12	180	8.88E-06
GO:0016604	nuclear body	24	742	1.08E-05
GO:0005635	nuclear envelope	18	446	1.48E-05
GO:0015630	microtubule cytoskeleton	30	1118	1.70E-05
GO:0044451	nucleoplasm part	29	1073	2.22E-05
GO:0031965	nuclear membrane	14	300	4.72E-05
GO:0005856	cytoskeleton	42	2068	0.00011
GO:1904813	ficolin-1-rich granule lumen	9	125	0.00011
GO:0044427	chromosomal part	23	819	0.00015
GO:0044430	cytoskeletal part	34	1547	0.00019
GO:0000785	chromatin	16	489	0.00059
GO:0071682	endocytic vesicle lumen	4	19	0.0009
GO:0001650	fibrillar center	8	136	0.0012
GO:0005681	spliceosomal complex	9	187	0.0018
GO:0060205	cytoplasmic vesicle lumen	12	340	0.0025
GO:0031967	organelle envelope	25	1146	0.0027
GO:0015030	Cajal body	5	57	0.0039
GO:0000793	condensed chromosome	9	215	0.0042
GO:0016581	NuRD complex	3	14	0.0059
GO:0005874	microtubule	12	385	0.0061
GO:0071013	catalytic step 2 spliceosome	6	99	0.0061
GO:0048471	perinuclear region of cytoplasm	16	670	0.0129
GO:0070545	PeBoW complex	2	4	0.0131
GO:0099512	supramolecular fiber	19	873	0.0131
GO:0012505	endomembrane system	62	4347	0.0138
GO:0030496	midbody	7	165	0.0141
GO:0005819	spindle	10	322	0.0151
GO:0098687	chromosomal region	9	272	0.0165
GO:0072686	mitotic spindle	5	87	0.0179
GO:0000228	nuclear chromosome	13	514	0.0189
GO:0000127	transcription factor TFIIIC complex	2	6	0.0205
GO:0044295	axonal growth cone	3	26	0.0222
GO:0044454	nuclear chromosome part	12	480	0.0283
GO:0005815	microtubule organizing center	15	683	0.0306
GO:0030686	90S preribosome	3	30	0.0306
GO:0042555	MCM complex	2	8	0.0306
GO:0042641	actomyosin	4	63	0.0311
GO:0031256	leading edge membrane	6	150	0.0325
GO:0031252	cell leading edge	10	371	0.0332

GO:1990391	DNA repair complex	3	32	0.0336
GO:0005844	polysome	4	66	0.034
GO:0035770	ribonucleoprotein granule	7	204	0.034
GO:0000346	transcription export complex	2	10	0.0388
GO:0034774	secretory granule lumen	9	323	0.0388
GO:0044449	contractile fiber part	7	212	0.0396
GO:0035861	site of double-strand break	3	36	0.0417
GO:0001726	ruffle	6	164	0.0422
GO:0005643	nuclear pore	4	72	0.0422
GO:0030016	myofibril	7	216	0.0422
GO:0030688	preribosome, small subunit precursor	2	11	0.0422
GO:0044297	cell body	12	526	0.0444
GO:0005813	centrosome	11	468	0.049
**Reactome Pathway**				
HSA-1640170	Cell Cycle	22	586	0.00012
HSA-8953854	Metabolism of RNA	21	652	0.0013
HSA-3371556	Cellular response to heat stress	7	85	0.0081
HSA-69278	Cell Cycle, Mitotic	16	483	0.0081
HSA-72203	Processing of Capped Intron-Containing Pre-mRNA	11	234	0.0081
HSA-3000484	Scavenging by Class F Receptors	3	6	0.009
HSA-168254	Influenza Infection	8	148	0.0167
HSA-68886	M Phase	12	343	0.0189
HSA-73894	DNA Repair	11	290	0.0189
HSA-3700989	Transcriptional Regulation by TP53	12	359	0.0241
HSA-3371511	HSF1 activation	3	12	0.0253
HSA-68884	Mitotic Telophase/Cytokinesis	3	14	0.0319
HSA-72163	mRNA Splicing - Major Pathway	8	178	0.0319
HSA-159236	Transport of Mature mRNA derived from an Intron-Containing Transcript	5	69	0.0333
HSA-1643685	Disease	22	1018	0.0333
HSA-168253	Host Interactions with Influenza Factors	4	39	0.0333
HSA-168276	NS1 Mediated Effects on Host Pathways	4	37	0.0333
HSA-2173782	Binding and Uptake of Ligands by Scavenger Receptors	4	40	0.0333
HSA-4615885	SUMOylation of DNA replication proteins	4	40	0.0333
HSA-5653656	Vesicle-mediated transport	16	649	0.0333
HSA-68877	Mitotic Prometaphase	8	190	0.0333
HSA-69481	G2/M Checkpoints	7	148	0.0333
HSA-72172	mRNA Splicing	8	186	0.0333
HSA-72312	rRNA processing	8	199	0.0333
HSA-8868773	rRNA processing in the nucleus and cytosol	8	189	0.0333
HSA-8953897	Cellular responses to external stimuli	13	459	0.0333
HSA-445144	Signal transduction by L1	3	20	0.034
HSA-5633007	Regulation of TP53 Activity	7	159	0.034
HSA-5663205	Infectious disease	11	363	0.034
HSA-72202	Transport of Mature Transcript to Cytoplasm	5	77	0.034
HSA-74160	Gene expression (Transcription)	26	1366	0.034
HSA-2262752	Cellular responses to stress	11	384	0.0441
HSA-8956321	Nucleotide salvage	3	23	0.0445
HSA-5693571	Nonhomologous End-Joining (NHEJ)	4	52	0.0472

Table S2. KEGG pathway enrichment analysis of motif-centric phosphorylated sites with multiple kinases using DAVID (related to Figure 6a).

Pathway	FDR	Count
RNA transport	8.87E-22	100
Spliceosome	4.60E-20	82
Biosynthesis of antibiotics	1.09E-11	96
Ribosome	1.82E-10	68
Carbon metabolism	5.04E-10	59
Protein processing in endoplasmic reticulum	3.91E-09	76
Ribosome biogenesis in eukaryotes	5.58E-08	46
Proteasome	1.37E-07	29
Biosynthesis of amino acids	3.97E-07	39
mRNA surveillance pathway	8.48E-07	45
DNA replication	1.31E-05	23
RNA degradation	3.46E-05	37
Cell cycle	8.17E-05	51
Alzheimer's disease	1.80E-04	63
Pyruvate metabolism	4.68E-04	22
Cysteine and methionine metabolism	6.49E-04	21
Mismatch repair	0.0011	15
Glycolysis / Gluconeogenesis	0.0014	30
Nucleotide excision repair	0.0022	23
Citrate cycle (TCA cycle)	0.0025	17
Oocyte meiosis	0.0036	42
Ubiquitin mediated proteolysis	0.0047	49
Huntington's disease	0.0049	64
Aminoacyl-tRNA biosynthesis	0.0053	28
Parkinson's disease	0.0053	50
Metabolic pathways	0.0074	316
ErbB signaling pathway	0.0135	33
Insulin signaling pathway	0.0154	47
Pentose phosphate pathway	0.0154	15
Viral carcinogenesis	0.0358	63
Oxidative phosphorylation	0.0358	44
Pathogenic Escherichia coli infection	0.0358	21
Epstein-Barr virus infection	0.0358	41
Fatty acid metabolism	0.0370	20
Endocytosis	0.0370	72

Table S3. The information on the peptide amount in each TMT channel for all experiments (related to Star Methods).

Figure	Experiment	TMT channel	Peptide amount	Cell condition	Kinase reaction	Raw files
Figure 2	Exp. 1	126	25 µg	DMSO	N.A.	170705Fe_DCKi_CK2_TMT6_1_MS3.raw
		127	25 µg	CKi-treated	N.A.	170705Fe_DCKi_CK2_TMT6_2_MS3.raw
		128	25 µg	Motif-centric	CK2	
		129	25 µg	DMSO	N.A.	
		130	25 µg	CKi-treated	N.A.	
		131	25 µg	Motif-centric	CK2	
Figure S2	Exp. 2	126	25 µg	DMSO	N.A.	170715Fe_F50_PKA_TMT6_1.raw
		127	25 µg	forskolin -treated	N.A.	170715Fe_F50_PKA_TMT6_2.raw
		128	25 µg	Motif-centric	PKA	170715Fe_F50_PKA_TMT6_3.raw
		129	25 µg	DMSO	N.A.	
		130	25 µg	forskolin -treated	N.A.	
		131	25 µg	Motif-centric	PKA	
Figure S3b	Exp. 3	126		Empty		170620Fe_3plex_noPV_MS2.raw
		127	25 µg	EGF-treated	N.A.	
		128	25 µg	EGF/Afatinib-treated	N.A.	
		129		Empty		
		130		Empty		
		131		Empty		
	Exp. 4	126		Empty		170620Fe_3plex_PV50_MS2.raw
		127	25 µg	EGF-treated	N.A.	
		128	25 µg	EGF/Afatinib-treated	N.A.	
		129		Empty		
		130	50 µg	PV-treated	N.A.	
		131		Empty		
	Exp. 5	126		Empty		170620Fe_3plex_PV100_MS2.raw
		127	25 µg	EGF-treated	N.A.	
		128	25 µg	EGF/Afatinib-treated	N.A.	
		129		Empty		
		130	100 µg	PV-treated	N.A.	
		131		Empty		
	Exp. 6	126		Empty		170620Fe_3plex_PV150_MS2.raw
		127	25 µg	EGF-treated	N.A.	
		128	25 µg	EGF/Afatinib-treated	N.A.	
		129		Empty		
		130	150 µg	PV-treated	N.A.	
		131		Empty		
Figure 3,	Exp. 7	126	25 µg	EGF-treated	N.A.	170915Fe_EGFAFA_PV200_MS3_F1.raw

Figure S3a, S3c		127	25 µg	EGF/Afatinib-treated	N.A.	170915Fe_EGFAFA_PV200_MS3_F2.raw
		128	25 µg	EGF-treated	N.A.	170915Fe_EGFAFA_PV200_MS3_F3.raw
		129	25 µg	EGF/Afatinib-treated	N.A.	170915Fe_EGFAFA_PV200_MS3_F4.raw
		130		Empty		170915Fe_EGFAFA_PV200_MS3_F5.raw
		131	25 µg	PV-treated	N.A.	
	Exp. 8	126	25 µg	EGF-treated	N.A.	170915Fe_EGFAFA_EGFR_MS3_F1.raw
		127	25 µg	EGF/Afatinib-treated	N.A.	170915Fe_EGFAFA_EGFR_MS3_F2.raw
		128	25 µg	EGF-treated	N.A.	170915Fe_EGFAFA_EGFR_MS3_F3.raw
		129	25 µg	EGF/Afatinib-treated	N.A.	170915Fe_EGFAFA_EGFR_MS3_F4.raw
		130		Empty		170915Fe_EGFAFA_EGFR_MS3_F5.raw
		131	25 µg	Motif-centric	EGFR	
	Exp. 9	126	25 µg	EGF-treated	N.A.	170915Fe_EGFAFA_SRC_MS3_F1.raw
		127	25 µg	EGF/Afatinib-treated	N.A.	170915Fe_EGFAFA_SRC_MS3_F2.raw
		128	25 µg	EGF-treated	N.A.	170915Fe_EGFAFA_SRC_MS3_F3.raw
		129	25 µg	EGF/Afatinib-treated	N.A.	170915Fe_EGFAFA_SRC_MS3_F4.raw
		130		Empty		170915Fe_EGFAFA_SRC_MS3_F5.raw
		131	25 µg	Motif-centric	SRC	
Figure 5, Figure S4a	Exp. 10	126	25 µg	EGF-treated	N.A.	170921Fe_EAfa_CK2_TMT6_1.raw
		127	25 µg	EGF/Afatinib-treated	N.A.	170921Fe_EAfa_CK2_TMT6_2.raw
		128	25 µg	Motif-centric	CK2	170921Fe_EAfa_CK2_TMT6_3.raw
		129	25 µg	EGF-treated	N.A.	
		130	25 µg	EGF/Afatinib-treated	N.A.	
		131	25 µg	Motif-centric	CK2	
	Exp. 11	126	25 µg	EGF-treated	N.A.	170921Fe_EAfa_CDK_TMT6_1.raw
		127	25 ug	EGF/Afatinib-treated	N.A.	170921Fe_EAfa_CDK_TMT6_3.raw
		128	25 ug	Motif-centric	CDK1	
		129	25 ug	EGF-treated	N.A.	
		130	25 ug	EGF/Afatinib-treated	N.A.	
		131	25 ug	Motif-centric	CDK1	
	Exp. 12	126	25 ug	EGF-treated	N.A.	170921Fe_EAfa_ERK_TMT6_1.raw
		127	25 ug	EGF/Afatinib-treated	N.A.	170921Fe_EAfa_ERK_TMT6_2.raw
		128	25 ug	Motif-centric	ERK2	
		129	25 ug	EGF-treated	N.A.	
		130	25 ug	EGF/Afatinib-treated	N.A.	
		131	25 ug	Motif-centric	ERK2	
	Exp. 13	126	25 ug	EGF-treated	N.A.	170921Fe_EAfa_JNK_TMT6_1.raw
		127	25 ug	EGF/Afatinib-treated	N.A.	170921Fe_EAfa_JNK_TMT6_2.raw
		128	25 ug	Motif-centric	JNK1	
		129	25 ug	EGF-treated	N.A.	
		130	25 ug	EGF/Afatinib-treated	N.A.	
		131	25 ug	Motif-centric	JNK1	
	Exp. 14	126	25 ug	EGF-treated	N.A.	170921Fe_EAfa_p38a_TMT6_1.raw
		127	25 ug	EGF/Afatinib-treated	N.A.	170921Fe_EAfa_p38a_TMT6_3.raw
		128	25 ug	Motif-centric	p38a	

		129	25 ug	EGF-treated	N.A.	
		130	25 ug	EGF/Afatinib-treated	N.A.	
		131	25 ug	Motif-centric	p38a	
	Exp. 15	126	25 ug	EGF-treated	N.A.	170921Fe_EAfa_EGFR_TMT6_1.raw
		127	25 ug	EGF/Afatinib-treated	N.A.	70921Fe_EAfa_EGFR_TMT6_2.raw
		128	25 ug	Motif-centric	EGFR	170921Fe_EAfa_EGFR_TMT6_3.raw
		129	25 ug	EGF-treated	N.A.	
		130	25 ug	EGF/Afatinib-treated	N.A.	
		131	25 ug	Motif-centric	EGFR	
	Exp. 16	126	25 ug	EGF-treated	N.A.	170921Fe_EAfa_SRC_TMT6_1.raw
		127	25 ug	EGF/Afatinib-treated	N.A.	170921Fe_EAfa_SRC_TMT6_2.raw
		128	25 ug	Motif-centric	SRC	170921Fe_EAfa_SRC_TMT6_3.raw
		129	25 ug	EGF-treated	N.A.	
		130	25 ug	EGF/Afatinib-treated	N.A.	
		131	25 ug	Motif-centric	SRC	
Figure 4	Exp. 17	126	5 ug	Un-treated	N.A.	210825ko02Motif_EGFR_Set1_1.raw
		127	10 ug	Un-treated	N.A.	210825ko02Motif_EGFR_Set1_2.raw
		128	25 ug	Un-treated	N.A.	210825ko02Motif_EGFR_Set1_3.raw
		129	60 ug	Un-treated	N.A.	
		130		Empty		
		131	5 ug	Motif-centric	EGFR	
	Exp. 18	126	5 ug	Un-treated	N.A.	210825ko02Motif_EGFR_Set2_1.raw
		127	10 ug	Un-treated	N.A.	210825ko02Motif_EGFR_Set2_2.raw
		128	25 ug	Un-treated	N.A.	210825ko02Motif_EGFR_Set2_3.raw
		129	60 ug	Un-treated	N.A.	
		130		Empty		
		131	25 ug	Motif-centric	EGFR	
	Exp. 19	126	5 ug	Un-treated	N.A.	210825ko02Motif_EGFR_Set3_1.raw
		127	10 ug	Un-treated	N.A.	210825ko02Motif_EGFR_Set3_2.raw
		128	25 ug	Un-treated	N.A.	210825ko02Motif_EGFR_Set3_3.raw
		129	60 ug	Un-treated	N.A.	
		130		Empty		
		131	125 ug	Motif-centric	EGFR	
