# Supplementary material for: Motif-centric phosphoproteomics to target kinase-mediated signaling pathways
Source: Cell Rep Methods. 2022 Jan 14;2(1):100138. doi: 10.1016/j.crmeth.2021.100138 (PMC9017188; doi:10.1016/j.crmeth.2021.100138)
Supplement: Document S2. Article plus supplemental information [file mmc2.pdf]

Cell Reports
Methods

Article

Motif-centric phosphoproteomics to target kinase-mediated signaling pathways

Graphical abstract

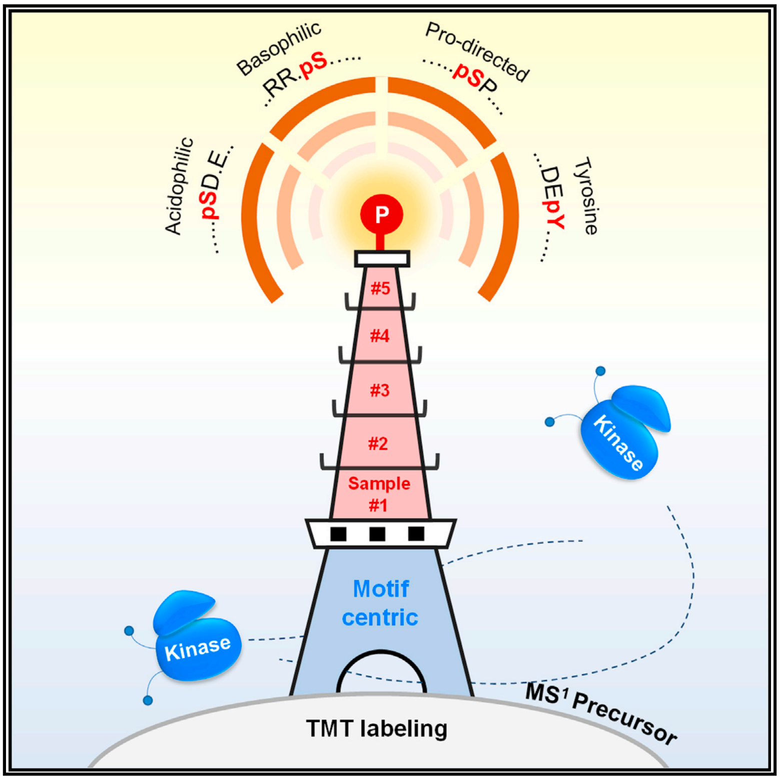

Authors

Chia-Feng Tsai, Kosuke Ogata, Naoyuki Sugiyama, Yasushi Ishihama

Correspondence

yishiham@pharm.kyoto-u.ac.jp

In brief

Tsai et al. present a motif-centric phosphoproteomics approach where *in vitro* kinase reactions are used to generate targeted phosphopeptides. These targeted phosphopeptides work as tandem mass tag carrier peptides to increase detectability and enable the quantification of thousands of pY sites from 25 μg of tryptic peptides as well as multiple targeted pathways.

Highlights

- Successful generation of motif-centric carrier peptides by *in vitro* kinase reactions
- Subsequent tandem mass tag quantification targeted to motif-centric phosphopeptides
- Successful quantitation of more than 7,000 pY sites from 25 μg of peptides achieved
- Multiple kinase-mediated pathways can be targeted for TMT quantitation

Tsai et al., 2022, Cell Reports Methods *2*, 100138
January 24, 2022 © 2021 The Author(s).
https://doi.org/10.1016/j.crmeth.2021.100138

CellPress

Cell Reports Methods

CellPress
OPEN ACCESS

Article

Motif-centric phosphoproteomics to target kinase-mediated signaling pathways

Chia-Feng Tsai,[1,3,4] Kosuke Ogata,[1,3] Naoyuki Sugiyama,[1] and Yasushi Ishihama[1,2,5,*]
[1]Graduate School of Pharmaceutical Sciences, Kyoto University, Kyoto 606-8501, Japan
[2]Laboratory of Clinical and Analytical Chemistry, National Institute of Biomedical Innovation, Health and Nutrition, Ibaraki, Osaka 567-0085, Japan
[3]These authors contributed equally
[4]Present address: Biological Sciences Division, Pacific Northwest National Laboratory, Richland, WA 99354, USA
[5]Lead contact
*Correspondence: yishiham@pharm.kyoto-u.ac.jp
https://doi.org/10.1016/j.crmeth.2021.100138

MOTIVATION Alterations in protein phosphorylation signaling networks associated with the dysregulation of kinase activity are closely linked to a variety of pathological conditions. However, quantitative and multiplexable assays to reveal the phosphorylation network based on kinase-substrate relationships do not exist for rare cell populations. To address this issue, we have developed an approach in which the signals of intrinsic kinase substrates are boosted using isobaric labeling that targets phosphopeptides generated in *in vitro* kinase reactions. As a result, we succeeded in encompassing specific kinase substrates in a motif-centric manner without the need for immunoprecipitation.

SUMMARY

Identifying cellular phosphorylation pathways based on kinase-substrate relationships is a critical step to understanding the regulation of physiological functions in cells. Mass spectrometry-based phosphoproteomics workflows have made it possible to comprehensively collect information on individual phosphorylation sites in a variety of samples. However, there is still no generic approach to uncover phosphorylation networks based on kinase-substrate relationships in rare cell populations. Here, we describe a motif-centric phosphoproteomics approach combined with multiplexed isobaric labeling, in which *in vitro* kinase reactions are used to generate targeted phosphopeptides, which are spiked into one of the isobaric channels to increase detectability. Proof-of-concept experiments demonstrate selective and comprehensive quantification of targeted phosphopeptides by using multiple kinases for motif-centric channels. More than 7,000 tyrosine phosphorylation sites were quantified from several tens of micrograms of starting materials. This approach enables the quantification of multiple phosphorylation pathways under physiological or pathological regulation in a motif-centric manner.

INTRODUCTION

Protein kinase-mediated phosphorylation on serine, threonine, and tyrosine residues is one of the most ubiquitous post-translational modifications (PTMs). Signaling cascades via protein phosphorylation play key roles in multiple cellular processes in mammals, including intra- and intercellular signaling, protein synthesis, gene expression, cell survival, and apoptosis (Cohen, 2002; Hunter, 2000; Needham et al., 2019). The relative abundances of phosphoserine (pS), phosphothreonine (pT), and phosphotyrosine (pY) sites in the human proteome have been estimated to be 90:10:0.05 based on the traditional method of ^{32}P labeling (Hunter and Sefton, 1980). There are many possible reasons for the extreme paucity of pY sites compared with pS and pT sites in mammals, including the fact that tyrosine kinases are activated only under certain conditions, and that the high activity of protein tyrosine phosphatase leads to a short half-life of pY sites (Hunter, 2009).

Great advances in the analytical workflows of shotgun phosphoproteomics, in which metal affinity chromatography is integrated with liquid chromatography-tandem mass spectrometry (LC-MS/MS), have made it possible to identify more than 30,000 phosphorylation sites (Hogrebe et al., 2018; Humphrey et al., 2015; Mertins et al., 2018). In general, LC-MS/MS has an identification bias toward the more abundant phosphopeptides in a sample, whereas in kinase substrates, sequence features such as Pro-directed, basophilic, acidophilic, and tyrosine-containing motifs have an important influence (Villen et al., 2007).

This is an open access article under the CC BY license (http://creativecommons.org/licenses/by/4.0/).

Also, since biological importance does not necessarily correlate with protein expression levels, it is possible that important signals are transduced via specific kinases that are expressed at extremely low levels. Therefore, advanced pre-fractionation or enrichment methods before LC-MS/MS are needed to identify a wide range of kinase substrates (Tsai et al., 2014). Especially for low-abundance pY peptides, MS detectability is affected by the ionization suppression caused by the presence of more abundant pS and pT peptides in the complex phosphoproteomes. However, the combination of metal affinity chromatography with immunoaffinity purification using a pY antibody (Abe et al., 2017) or a recently developed SH2 domain-derived pY-superbinder (Bian et al., 2016; Dong et al., 2017) has been reported to increase the identification of pY peptides. In addition, immunoaffinity-based methods using multiple antibodies have been developed for the identification and quantitation of phosphopeptides derived from proteins in various pathways or pY peptides derived from tyrosine kinases (Stokes et al., 2012). Nevertheless, a large amount of starting material (1–10 mg) is generally necessary for deep tyrosine phosphoproteome analysis.

One of the major advantages of multiplexed isobaric tandem mass tag (TMT)-based methods for relative quantitation is that the differentially labeled peptides appear as a single peak at the MS1 level (Thompson et al., 2003), enhancing the detectability of low-abundance peptides. TMT strategies using a large amount of relevant "boosting" (or "carrier") peptides labeled with one or several TMT channels have been successfully used for single-cell proteomics analysis (Budnik et al., 2018; Dou et al., 2019; Tsai et al., 2020). For instance, Yi et al. (2019) developed a Boosting to Amplify Signal with Isobaric Labeling (BASIL) strategy to quantify more than 20,000 phosphorylation sites in human pancreatic islets. However, the identification number of pY peptides was less than 1%. Recently, Chua et al. developed a Broad-spectrum Optimization Of Selective Triggering (BOOST) method, in which pervanadate (a tyrosine phosphatase inhibitor)-treated cells were used as a boosting channel to increase the detectability of pY peptides (Chua et al., 2020). The BOOST method coupled with antibody-based pY enrichment could quantify more than 2,300 unique pY peptides. However, the required amount of starting material was in the milligram range, making it difficult to apply to small samples such as clinical specimens, which often contain less than 100 μg of extractable material.

We previously developed an LC-MS/MS-based *in vitro* kinase assay using dephosphorylated lysate proteins as the substrate source for *in vitro* kinase reactions to profile human protein kinomes (Imamura et al., 2014). A total of 175,574 direct kinase substrates were identified from 354 wild-type protein kinases, 21 mutant protein kinases, and 10 lipid kinases (Sugiyama et al., 2019). In addition, we used the *in vitro* kinase reactions with CK2, MAPK, and EGFR to generate phosphopeptides with targeted motifs to measure the phosphorylation stoichiometry of more than 1,000 phosphorylation sites, including 366 low-abundance tyrosine phosphorylation sites (Tsai et al., 2015).

In the present study, we aimed to develop a motif-centric TMT approach in which phosphopeptides having targeted sequence motifs are generated by *in vitro* kinase reactions for the boosting TMT channel to increase the detectability of kinase substrates, including tyrosine kinase substrates, without immunoaffinity enrichment. To demonstrate the feasibility of this strategy, phosphopeptides with targeted motifs of CK2, PKA, CDK1, ERK2, JNK1, p38a, SRC, and EGFR were used for the boosting TMT channel to monitor the perturbation of kinase-mediated signaling pathways by tyrosine kinase inhibitor treatment.

RESULTS

Workflow for isobaric motif-centric phosphoproteome analysis

We previously developed a motif-centric approach (Tsai et al., 2015) in which dephosphorylation and isotope tagging are integrated with *in vitro* kinase reactions to improve the sensitivity and reproducibility for determining the absolute phosphorylation stoichiometry of targeted kinase substrates. However, the number of phosphosites commonly identified in endogenous and motif-centric phosphopeptides was not as large as expected, because some endogenous signals with specific kinase motifs are below the detection limit. In this study, we developed a motif-centric approach in which the same peptides from different samples were labeled with multiplexed TMT reagents and assembled as a single peak at the MS^1 level to increase the sensitivity. In addition, we set one of the TMT channels for signal boosting, using phosphopeptides having targeted sequence motifs generated by *in vitro* kinase reactions to increase the detectability of targeted kinase substrates. The entire workflow is shown in Figure 1. The motif-centric peptides are generated by *in vitro* kinase reactions using Pro-directed, acidophilic, basophilic, or tyrosine kinase (Figure 1A). The same biological resource (same cell type or tissue) can be used to accomplish the back-phosphorylation (Li et al., 2016; Mundina-Weilenmann et al., 1991) without any pre-dephosphorylation process(Tsai et al., 2015). *In vitro* kinase reactions both at the protein and tryptic peptide levels can be used in most cases, except for basophilic kinase reactions, where tryptic peptides cannot be used as substrates owing to the lack of K or R at the N-terminal side of the phospho accepting sites. In such a case, the kinase reactions at the protein level, followed by tryptic digestion, is used to generate the basophilic motif-centric phosphopeptides. After TMT labeling, phosphopeptides are enriched by immobilized metal ion affinity chromatography (IMAC) and analyzed by nanoLC-MS/MS. We recently reported that TMT-labeled phosphopeptides tend to pass through TiO_2 columns (Ogata et al., 2021), and the same phenomenon was also observed in IMAC system (Figure S1). Therefore, the IMAC protocol has been modified to increase the recovery of TMT phosphopeptides by decreasing the concentration of acetic acid in the loading buffer from 6% to 0.5%. We used this modified TMT-IMAC protocol for all experiments unless otherwise noted.

In tandem MS, the TMT-labeled precursor ions from the endogenous and back-phosphorylated peptides are fragmented and the assembled signals in b or y ions are helpful for peptide identification, especially for endogenous phosphopeptides with low abundance (Figure 1B). The relative quantitation of endogenous phosphopeptides across different samples is done by the use of reporter ions at the MS^3 level, by the implementation of

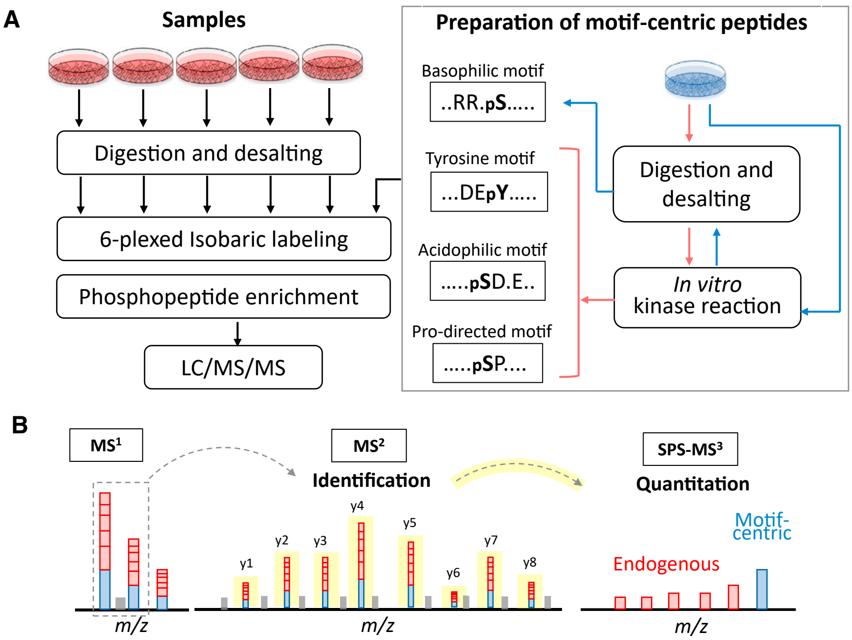

Figure 1. Workflow of isobaric motif-centric phosphoproteomics with *in vitro* kinase reactions

(A) The motif-centric peptides were generated by *in vitro* kinase reactions. Tryptic peptides from study samples and motif-centric peptides were labeled with different isobaric tags (TMT in this study). After mixing, the TMT-labeled phosphopeptides were enriched and analyzed by LC-MS/MS.

(B) TMT-labeled ions from endogenous and motif-centric phosphopeptides are assembled as a single peak at the MS^1 level and then fragmented at the MS^2 level to identify the peptide sequence. Relative quantification of endogenous phosphopeptides between different samples is achieved by reporter ions at the MS^3 level.

synchronous precursor selection (SPS)-based MS^3 technology (McAlister et al., 2014), which can decrease the interference signals owing to co-isolation of precursor ions.

Acidophilic motif-centric phosphoproteome analysis

We first used the motif-centric TMT approach to quantify the kinase-perturbed phosphorylation changes in HeLa cells using CK2 kinase inhibitor (CKi, Silmitasertib, CX-4945). The tryptic peptides from CKi-treated HeLa cells were phosphorylated by CK2 *in vitro* and labeled with both TMT^{128} and TMT^{131} for CK2 motif-centric boosting channels. The phosphopeptides from DMSO-treated cells were labeled with both TMT^{126} and TMT^{129}, and phosphopeptides from CKi-treated cells were labeled with both TMT^{127} and TMT^{130}. An MS^2 spectrum and an MS^3 spectrum of a known CK2 substrate, the pS66 site in LIG1, are shown in Figure 2. After fragmentation by collision-induced dissociation, the peptide sequence and phosphosite localization information can be annotated at the MS^2 level (Figure 2A). Then, the MS^3 spectrum was obtained, demonstrating that the TMT signals of endogenous phosphopeptides were decreased after the CKi treatment (TMT^{127} and TMT^{130} in Figure 2B) and that the TMT signals of CK2-motif targeting phosphopeptides were increased after *in vitro* kinase reactions (TMT^{128} and TMT^{131} in Figure 2B). These results indicate that this isobaric motif-centric approach can be used to monitor CK2 phosphorylation signaling in terms of the TMT ratios.

In total, this isobaric motif-centric approach quantified 4,559 unique phosphopeptides (91% specificity in phosphopeptide enrichment) from 25 μg of peptides per channel. The logarithm of the median ratio of CKi-treated to untreated peptides was negative, while the logarithm of the median ratio of motif-centric (CK2-motif) to CKi-treated peptides was positive and this increase was larger than the decrease caused by CKi treatment (Figure 2C), indicating that the *in vitro* kinase reactions effectively increased the TMT signals of peptides directly phosphorylated by CK2. Silmitasertib has used to exclusively inhibit CK2 activity in previous studies(Chon et al., 2015; Wang et al., 2017). Therefore, we can discriminate direct CK2 substrates from others by comparison of TMT ratios such as CKi/DMSO and CK2-motif/CKi using the Student *t* test. Based on the CKi/DMSO ratios, 66 (1.6%) up-regulated and 750 (18%) down-regulated phosphorylation sites were identified (Figure 2D). Among the down-regulated phosphorylation sites, up to 35% (n = 266) were significantly increased after *in vitro* kinase reactions (group B in Figure 2D). After sequence motif analysis (O'Shea et al., 2013), we found that the sequence motif of the phosphopeptides in group B agreed with the known CK2 substrate motif (acidic motif), whereas the motif logo from group A contained both Pro and Asp at the +1 position (Figure 2D). Among the phosphorylation sites (n = 266) in group B, 222 sites were registered in a public phosphorylation sites database (Hornbeck et al., 2015), including six known CK2 substrates: PTGES3 (S113), LIG1 (S66), SLC3A2 (S375), CDK1 (S39), TOP2A (S1377), and ABCF1(S110). For group B proteins, we also examined the overlap with CK2 interactors in STRING and found 20 known CK2 interactors (Figure 2E). We further performed Gene Ontology and Reactome Pathway enrichment analysis for group B proteins (Szklarczyk et al., 2019). As a result, 137 proteins in group B were annotated as nuclear proteins, and a majority of them possessed functions related to ATP binding, DNA binding, nucleotide binding and so on (Table S1). Among the annotated pathways, the top hit was the cell cycle pathway, in which 21 group B proteins were down-regulated, including CDK1, a known CK2 substrate that affects cell cycle regulation upon S39 phosphorylation (Bloom and Cross, 2007; Russo et al., 1992) (Figure 2F). All these results indicate that this motif-centric approach is an effective tool for monitoring specific kinase-mediated signaling pathways.

Basophilic motif-centric phosphoproteome analysis

The isobaric motif-centric approach was further examined with PKA as a basophilic kinase, using forskolin as an activator. Unlike acidophilic CK2, PKA cannot be used to generate

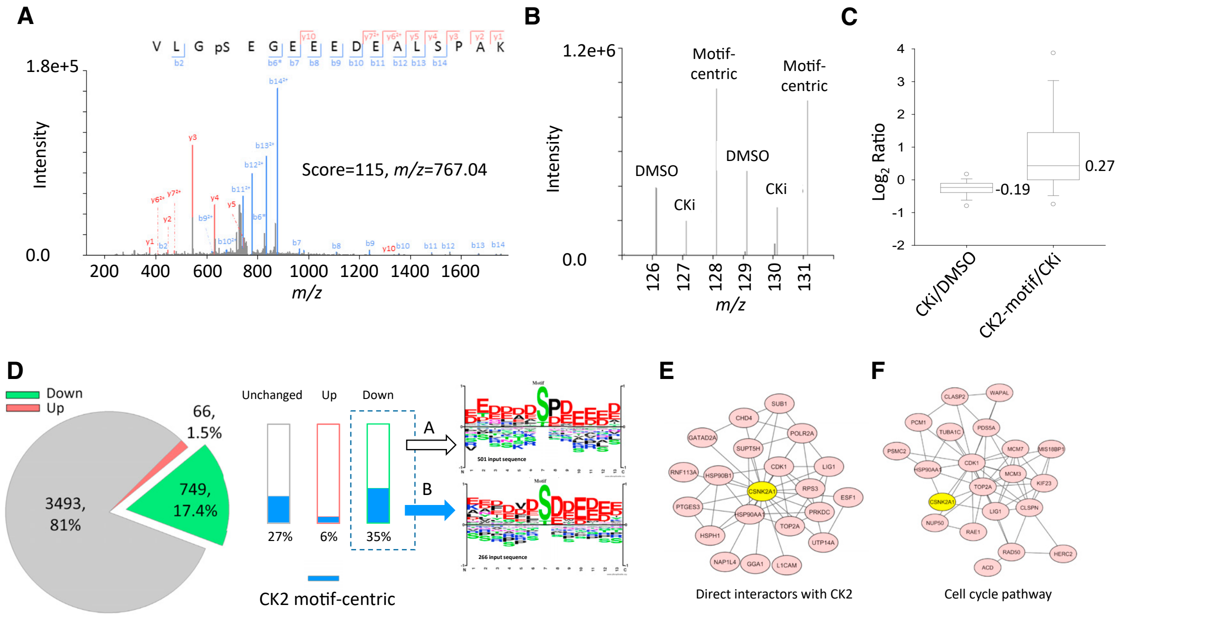

Figure 2. Typical example of the isobaric CK2 motif-centric phosphoproteomic approach

(A) An MS/MS spectrum of TMT-labeled phosphopeptide (V L Gp S E G E E E D E A L S P A K, triply charged). Product ions at the MS^2 level identified the peptide sequence and phosphorylation site localization.

(B) An MS/MS/MS spectrum at the 6-plexed TMT reporter ion region. Three samples (DMSO, CKi-treated, and CK2 motif-centric phosphopeptides) in duplicate preparations, labeled with 6-plexed TMT reagents, were quantified at the MS^3 level.

(C) The ratio distribution on a log_2 scale of identified phosphopeptides. Left bar: CKi/DMSO, right bar: motif-centric/CKi.

(D) Quantitation result for CKi treatment. The red and green parts in the pie chart mean up-regulated and down-regulated phosphorylation sites after CKi treatment, respectively. The blue bar indicates the amount of phosphorylation sites significantly increased after *in vitro* kinase reactions (CK2-motif centric peptides). Sequence motif analysis of the down-regulated phosphorylation sites after CKi treatment was performed for CK2-motif centric peptides (group B) and other peptides (group A).

(E) Protein-protein interaction analysis by STRING. Twenty proteins were identified as overlapped proteins between CK2 direct interactors in STRING and group B proteins.

(F) Proteins in the cell cycle pathway identified by REACTOME pathway enrichment analysis of group B proteins. Duplicate sample preparation and duplicate LC-MS/MS analyses were performed for each TMT set as shown in Table S3.

motif-centric peptides from tryptic peptides because it requires basic amino acid residues at the N-terminal side of the phosphoacceptor. Therefore, PKA motif-centric peptides were generated by *in vitro* kinase reactions at the protein level, followed by tryptic digestion (Figure 1A). After the isobaric labeling, the TMT ratios of three channels, forskolin-treated, DMSO-treated, and motif-centric samples, were used to discriminate the peptides phosphorylated directly by PKA from others, as in the case of CK2. The results are illustrated in Figure S2. In total, we quantified 7,855 phosphorylation sites from biological replicate experiments. Among them (Figure S2A), 620 phosphorylation sites were up-regulated (group 1) and 322 phosphorylation sites were inhibited by forskolin (group 2). We previously established a computational model (primary sequence preference [PSP score]) to characterize the kinase sequence specificity toward the substrate target site based on known kinase-substrate relationships, to exclude potential indirect targets of PKA (Imamura et al., 2017). We applied PSP scoring to the phosphorylation sites in groups 1 and 2, and found that the PSP scores of group 1 were significantly higher than those of group 2, although the sequence motifs of phosphorylation sites in groups 1 and 2 both belonged to the basophilic category (Figure S2B). Based on the above results, the motif-centric approach was able to discriminate peptides phosphorylated directly by PKA from indirectly phosphorylated ones.

Tyrosine motif-centric phosphoproteome analysis

Although the detectability of pY sites has been restricted by their extremely low phosphorylation stoichiometry compared with pS and pT sites(Sharma et al., 2014; Tsai et al., 2015), this lower stoichiometry results in a larger amount of unphosphorylated counterparts, which can be used for back-phosphorylation. To increase the identification number of pY sites, we firstly tried to use pervanadate (PV)-treated HeLa cells as a pY-centric sample (Figure S3A). PV is well-known as a tyrosine phosphatase inhibitor causing an increase in the stoichiometry of endogenous pY sites (Sharma et al., 2014). As expected, the pY content was increased from 0.8% to 11.7% (Figure S3B) by PV treatment. Then we used the PV-treated peptides as pY-centric peptides with 25 μg of untreated HeLa peptides. Indeed, the number of quantifiable pY sites increased from 74 to 595 as the spiking amount of the pY-centric peptides was increased from 0 to 150 μg, without any antibody-based enrichment (Figure S3B). The amplified signals caused by the spiked pY peptides resulted

CellPress
OPEN ACCESS

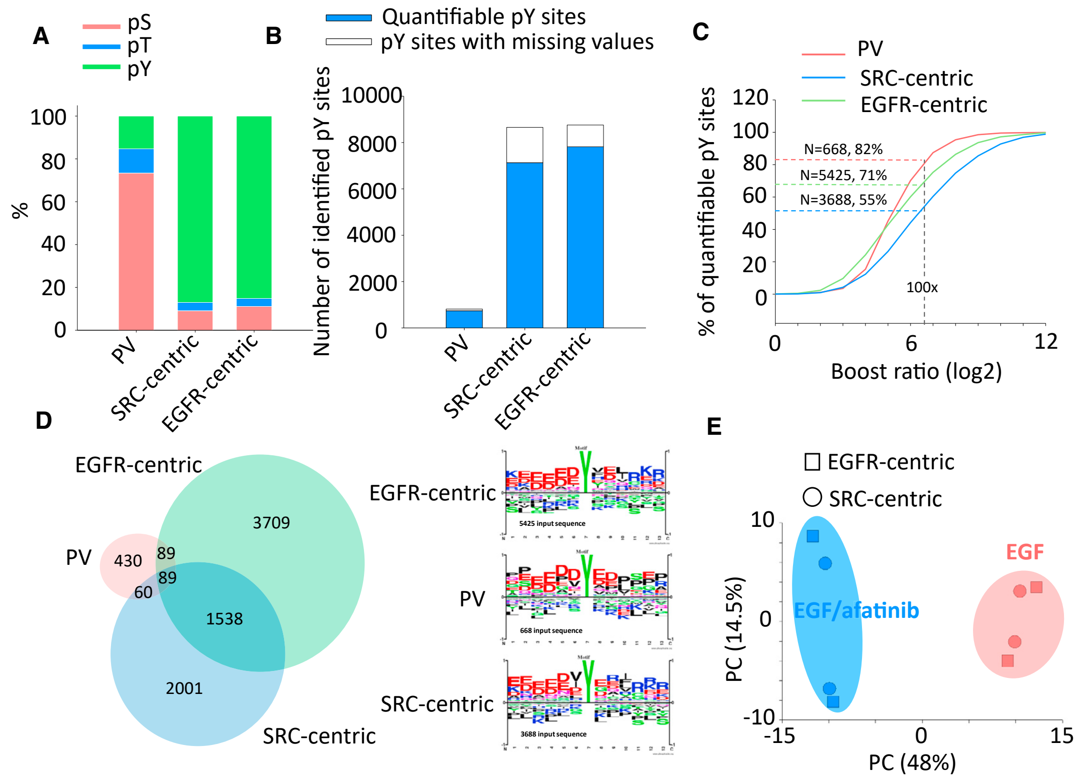

Figure 3. Summary of pY motif-centric phosphoproteomic approach

(A) The content (%) of identified pY sites using PV-treated, SRC and EGFR phosphorylated peptides as the pY-centric samples.

(B) The number of pY sites identified in each experiment. A quantifiable site is defined as a pY site having signal intensities in at least 2 TMT reporter channels. Otherwise, pY sites are considered as missing values.

(C) The content of quantifiable pY sites filtered by boost ratio, defined as the TMT signal at *m/z* 131 divided by the averaged signal at *m/z* 127 and 129.

(D) The overlap and the sequence logos of boost ratio-filtered quantified pY sites between different motif-centric approaches

(E) The principal component analysis of commonly quantified pY sites (filtered). Duplicate sample preparation with one LC-MS/MS analysis with a long silica monolith column were performed for each TMT set as shown in Table S3.

in a much larger number of identified pY sites than in usual phosphoproteome analysis. However, the number of identified pY sites was still lower than that obtained using the recently published antibody-based boosting strategy (Chua et al., 2020), which also used PV-treated cells as boosting samples to quantify more than 2,300 unique pY peptides from 1 mg of starting materials.

To extend the tyrosine phosphoproteome coverage, the back-phosphorylation sites of the untreated HeLa peptides were phosphorylated via *in vitro* kinase reactions by using tyrosine kinases such as EGFR and SRC. Note that EGFR and SRC have different phosphorylation motifs (Sugiyama et al., 2019). Then, the kinase-treated peptides were labeled with one of the 6-plexed TMT reagents (each TMT channel contained 25 μg tryptic peptides), to detect the endogenous pY sites in EGF or EGF/afatinib (EGFR inhibitor)-treated HeLa cells (Figure S3A). After TMT labeling followed by IMAC enrichment, the TMT-labeled endogenous phosphopeptides mixed with PV treated, EGFR-centric or SRC-centric phosphopeptides were analyzed on a 2-m-long monolithic silica column system with the SPS-MS^3 technique (McAlister et al., 2014). Compared with the result from PV-treated HeLa as pY motif-centric peptides, the content of pY increased from 15% to 87% (Figure 3A), which resulted in an approximately 10-fold increase in the numbers of identified and quantified class 1 pY sites (localization probability >0.75), as shown in Figure 3B. However, Cheung et al. (2021) reported that the isobaric labeling-based quantitative approaches have technical limitations that potentially affect data quality and biological interpretation, owing to the large amounts of spiked carrier samples. In addition, it is difficult to control the TMT ratio within the quantifiable range because the phosphorylation stoichiometry in cells depends on each pY site. Furthermore, we should reject TMT peptides without reporter ion signals in the sample channels. Therefore, we examined the distribution of the TMT reporter ion intensity of each sample channel and found a notch to discriminate the signal from the noise (Figure S3C); this was also mentioned in the previous study (Hughes et al., 2017). Based on this observation, we set the acceptance criterion for the minimum TMT intensity in the sample channels to be greater than 40 on a $\log_2$ scale for the total TMT intensity of the sample channels. We also set another criterion—that the maximum TMT ratio of the motif-centric channel to the sample channel should be less than 100 (Figure 3C)—to minimize the ion sampling effect (Cheung et al., 2021; Tsai et al., 2020). By

applying these two criteria, we found that 668, 5,425, and 3,688 pY sites could be quantified in the PV-treated, EGFR-centric, and SRC-centric samples, respectively. Among these pY sites, the sequence motifs of unique pY sites in each dataset were different (Figure 3D), which indicated that complementary pY sites can be identified by using different motif-centric peptides. For quantitation based on the TMT reporter ion intensities, the EGF and EGF-afatinib treated cells were separated in the principal component analysis (Figure 3E). The above results demonstrate that this motif-centric approach through spiking isobaric pY peptides with high purity was able to increase the detectability of pY sites without the need for pY-specific affinity purification, such as using pY antibodies.

We further evaluated the spiking effect of the carrier/boost amount of motif-centric peptides on the number of quantifiable phosphosites. Different amounts of TMT-labeled peptides (5, 10, 25, and 60 μg) from untreated HeLa cells were mixed with different amounts (5, 25, and 125 μg) of pY motif (EGFR)-centric peptides (Figure 4A). In these experiments, we applied a nanoscale solid phase TMT labeling protocol to phosphopeptides enriched by TiO_2 chromatography to accommodate smaller amounts of peptides (Ogata et al., 2021). The distribution of the quantifiable pY site fraction for different boost ratios is shown in Figure 4B. When we applied the acceptance criterion that the boost ratio should be less than 100, a higher fraction of pY sites was obtained as we increased the amount of endogenous peptides with the constant amount of motif-centric peptides. The same tendency was observed for the number of quantifiable pY sites (Figure 4C). As the amount of the endogenous peptides increased, the number of quantifiable pY sites increased linearly, regardless of the amount of motif-centric peptides. In contrast, when the amount of the endogenous peptides was limited, the number of quantifiable pY sites were saturated or even decreased with increasing amounts of motif-centric peptides. Based on these results, in this study, the peptide amount per TMT channel was set to 25 μg for both endogenous and motif-centric channels.

Multiple motifs-centric approach to depict perturbed phosphoproteome

We further used multiple kinases to generate a wide variety of motif-centric peptides to monitor the phosphorylation signals generated by different kinases. For proof-of-concept, we analyzed the phosphoproteome of EGF-treated and EGF/afatinib co-treated HeLa cells. We selected four Pro-directed kinases (ERK1 [MAPK3], JNK1 [MAPK8], p38α [MAPK14] and CDK1), two tyrosine kinases (SRC and EGFR), and one acidophilic kinase (CK2) to detect the different motif-centric phosphorylation sites (Figure S4A). After applying the following acceptance criteria—the boost ratio should be less than 100, the total TMT intensity of the sample channels must be greater than 40 on a log_2 scale, and two valid TMT values must be obtained in at least one of duplicate channels—up to 11,895 class 1 phosphorylation sites were quantified, including 5,045 pS, 1,756 pT, and 5,094 pY sites (Figure 5A). Of the 11,895 quantifiable class 1 phosphorylation sites, up to 8,482 were quantified as motif-centric phosphopeptides, indicating that endogenous peptides that are not motif-centric do not interfere with the quantification of the target phosphopeptides under the current conditions. According to the Student *t* test (EGF vs. EGF/afatinib), the ratio of commonly regulated phosphorylation sites was consistent between experiments using different kinases, regardless of the kinase used (Figure 5B), indicating that the boosting channel did not significantly affect the quantitative results.

In addition to identifying which phosphorylation sites were regulated after afatinib treatment, we were also able to estimate which kinases phosphorylated which sites by using this motif-centric approach. For example, up to 18 class 1 phosphorylation sites, including 7 pY sites, on EGFR kinase were quantified without any immunoprecipitation step from 25 μg of starting materials per TMT channel (Figure 5C). Among these sites, pY1144 is a known autophosphorylation site which was down-regulated by afatinib and detected in the EGFR motif-centric experiment. This result indicates that the activity of this phosphorylation site is controlled by EGFR and also inhibited by afatinib. In contrast, pS1051 and pS992 were down-regulated by afatinib, but there is no information about the corresponding kinase in the public database. Through this motif-centric approach, we could determine that pS1051 and pS992 are likely phosphorylated by CK2 and ERK2, respectively.

Another advantage of the motif-centric approach is the specificity of the *in vitro* kinase reactions. The Student *t* test (EGF/afatinib vs. motif-centric) revealed a low overlap of motif-centric peptides among the kinases (Figure 5D). Although ERK2, JNK1, p38a, and CDK1 are all Pro-directed kinases, complementary profiles for the quantified motif-centric phosphorylation sites were observed (Figure 5D). The motif logos of these sites showed slight differences among the four Pro-directed kinases (Figure S4B). For example, the proportion of pT motifs was higher with p38α kinase. In addition, more acidic amino acids were located at the C-terminal side of the phospho-acceptor site in the case of ERK2 kinase compared with CDK1 kinase. In addition to S/T sites, the pY motifs also differed between EGFR- and SRC-centric phosphopeptides (Figure S4C).

Furthermore, from the two ratios (EGF vs. motif-centric and EGF/afatinib vs. motif-centric), we can estimate the stoichiometry of motif-centric phosphorylation sites (Figure 5E), assuming that the efficiency of *in vitro* kinase reactions is 100%. The phosphorylation sites containing acidophilic kinase substrates targeted by CK2 generally exhibited higher phosphorylation stoichiometry than sites targeted by Pro-directed kinase and tyrosine kinases. The phosphorylation stoichiometry distribution that we observed here is consistent with our previous findings (Tsai et al., 2015). To further validate that the endogenous signals boosted by motif-centric peptides are lower than other peptide signals, the peak areas in the XICs of MS1 signals of quantified phosphopeptides were calculated based on the proportion of TMT intensity (Figures S4D and S4E). The XICs of endogenous phosphopeptides boosted by the motif-centric peptides were lower than those of other phosphopeptides, which indicates the motif-centric approach is effective to identify these low-abundance kinase substrates.

In this motif-centric approach, a specific kinase can be chosen to target an endogenous peptide that is phosphorylated by that kinase. To confirm this, 8,482 motif-centric phosphopeptides prepared with these seven kinases were subjected to KEGG

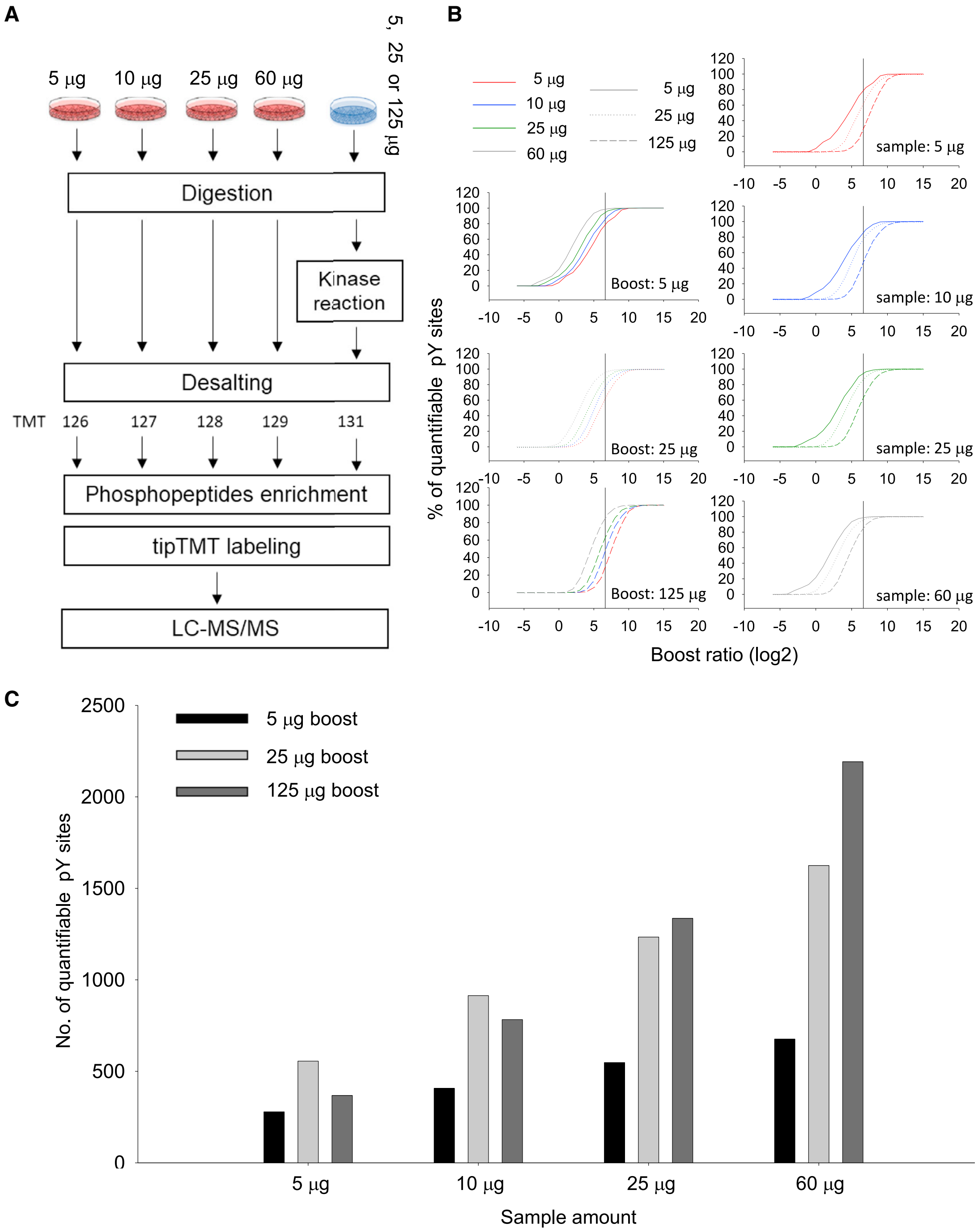

Figure 4. Effects of the boost ratio of spiking motif-centric peptides on quantifiable pY sites

(A) Different amount of TMT labeled peptides from un-treated HeLa cell (5, 10, 25, and 60 μg) were mixed with different amount (5, 25, and 125 μg) of pY motif (EGFR)-centric peptides. After phosphopeptides enrichment, the TMT-labeled phosphopeptides were analyzed by LC-MS/MS.

(B) The distribution of the quantifiable pY site fraction for different boosting ratios.

(C) The number of detected pY sites under different amount of motif-centric peptides (boost ratio <100×). Triplicate sample preparation of 4 different amount of HeLa peptides with three different amounts of pY motif-centric peptides was performed and triplicate LC-MS/MS analyses were performed for each TMT set as shown in Table S3.

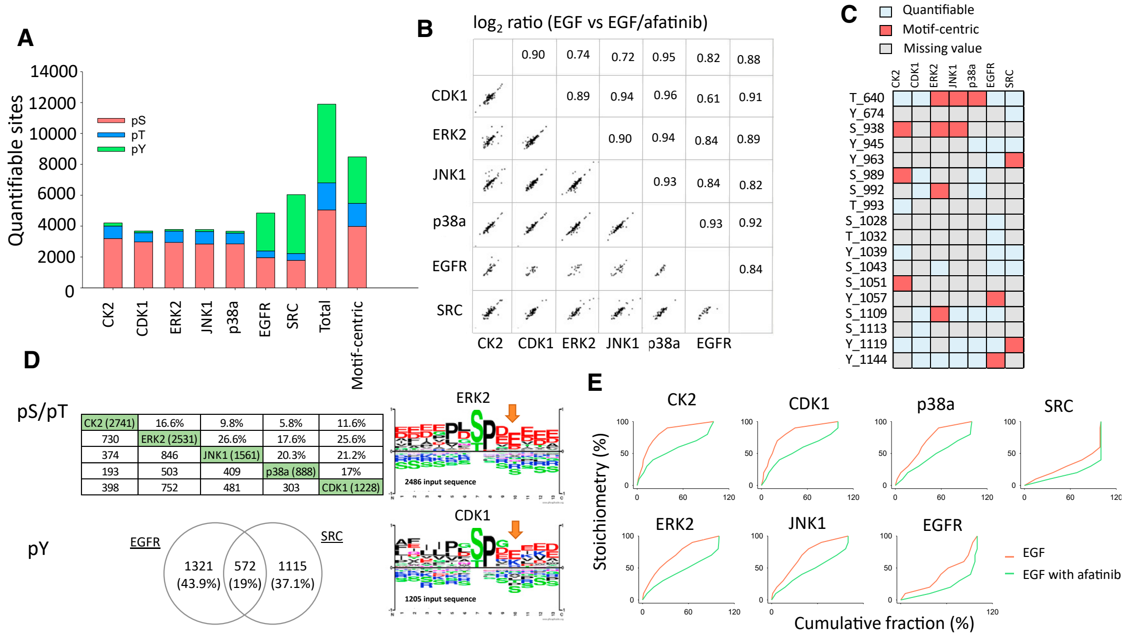

Figure 5. Summary of motif-centric phosphoproteomic approach with multiple kinases

(A) The number of quantified phosphorylation sites (class 1) obtained using different kinases.

(B) The ratio correlation of commonly regulated phosphorylation sites (EGF vs EGF/afatinib, $p < 0.05$) in experiments with different kinases.

(C) The motif-centric sites (red color) among quantified phosphorylation sites in EGFR.

(D) The overlap of motif-centric phosphorylation sites between the samples with different kinases as motif-centric samples (left), and the sequence motif analysis for the ERK2 and CDK1 centric phosphorylation sites (right).

(E) The phosphorylation stoichiometry distribution of motif-centric phosphorylation sites with 10 μM EGF (red) and 10 μM EGF/10 μM afatinib (green) treatment. The stoichiometric values of the phosphosites were calculated from the ratios of the signal in the endogenous peptide channel to the signal in the motif-centric back phosphorylation channel. After sorting the stoichiometric values in ascending order, the cumulative fraction percent and the corresponding stoichiometry were then calculated. Duplicate sample preparation and two or three replicate LC-MS/MS analyses were performed for each kinase as shown in Table S3.

pathway enrichment analysis using DAVID (Huang da et al., 2009). As a result, known pathways involving the seven kinases, such as ErbB and insulin signaling, were enriched as expected (Figure 6A and Table S2). We then mapped the responsible kinases for the *in vitro* phosphorylation sites on the 33 phosphoproteins comprising the ErbB pathway identified in this enrichment analysis (Figure 6B), showing how the kinases used for motif-centric peptides covered the targeted phosphosites within the targeted pathway. In other words, we can manipulate the targeted pathway by choosing the appropriate kinases, using our large-scale library of *in vitro* kinase-substrate relationships (Sugiyama et al., 2019). Overall, our findings indicate that the motif-centric approach can provide system-wide customizable maps consisting of targeted pathways under physiological or pathological regulation.

DISCUSSION

Previously, the kinase assay-linked phosphoproteomics approach (Xue et al., 2012, 2013) has been developed to find potential kinase substrates which were found in the overlap of the *in vitro* phosphopeptides generated by kinase reactions with dephosphorylated peptides and *in vivo* kinase-dependent phosphorylation events in different LC-MS/MS runs. However, the dephosphorylation is not complete, and this may result in the false-positive identification of putative kinase substrates. In addition, the analytical throughput is still limited by the need for multiple LC-MS/MS analysis, and the background effect owing to incompletely dephosphorylated peptides decreases the detection sensitivity for putative kinase substrates. To tackle these challenges, the isobaric motif-centric approach developed in this study can link *in vitro* substrates and physiological phosphorylation events by monitoring both endogenous and back (specific kinase motif-targeting) phosphorylated signals in a single LC-MS/MS run without the need for a further dephosphorylation step, thereby enabling the high-throughput analysis of putative kinase substrates.

Unlike metal affinity chromatography for enrichment of phosphopeptides, motif-specific immunoaffinity precipitation (IAP)-based LC-MS/MS makes it possible to recognize a characteristic sequence motif from a broad range of peptides by using different motif antibodies. Because each antibody binds phosphopeptides followed by a specific peptide motif, the overlap of identified phosphopeptides among different antibodies is low. The results of this approach are similar to those obtained with our motif targeting approach (Figure 4D), in which recognition between kinase and peptides is based on the specific sequence motif (Stokes et al., 2015; Sugiyama et al., 2019). The overlap between a given antibody and metal affinity chromatography ranged from roughly 16% with the all Ser/Thr antibody mix, to a low of only 3.6% with pY-1000 (Stokes et al., 2015).

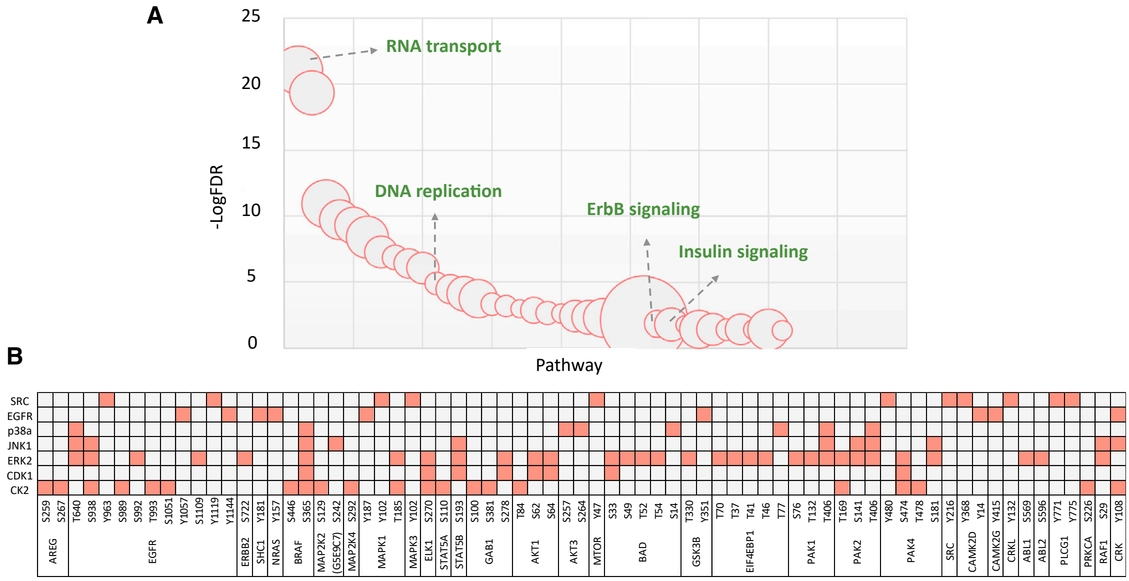

Figure 6. Pathway enrichment analysis of motif-centric phosphorylated sites with multiple kinases

(A) KEGG pathway enrichment analysis by DAVID.

(B) Phosphorylation sites on proteins enriched in the ErbB signaling pathway and the corresponding kinases.

Anthony et al. aimed to enlarge the phosphoproteome coverage by using both TiO_2 followed by basic pH reversed-phase fractionation and motif-specific IAP with four different phosphorylation motif-specific antibodies (Possemato et al., 2017). In total, 8,947 nonredundant peptides were identified in the TiO_2 dataset, of which only 852 (9.5%) were in common with the peptides identified in the IAPs. These results suggest that the range of phosphorylation within a given system is so broad that no single approach is likely to provide comprehensive coverage.

In the case of IAPs, the specificity of the antibodies is not high enough to distinguish phosphopeptides with similar motifs; for example, MAPK phosphorylates substrates with the consensus sequence PX(S/T)P and CDKs phosphorylate substrates containing the consensus sequence (S/T)PXR/K (Songyang et al., 1996). However, the complementary regulated motif-centric phosphorylation sites (Figures 5D and S4B) were differentiated in this study, even though the kinases all belong to the same Pro-directed kinase group. The motif-centric approach was also able to distinguish the motif difference (PX(S/T)P and (S/T)PXR/K) for ERK2 and CDK1 (Figure 5D). In addition, the purification specificity in IAP (<50%) is much lower than that in the metal affinity-based approach (Possemato et al., 2017), which means that milligram amounts of starting materials are necessary for the IAPs method. For our isobaric motif-centric approach, we obtained a purification specificity of more than 90% (Figure S4F). In addition, the IAPs approach uses selected antibodies to isolate targeting peptides, and this may cause unnecessary sample loss during the purification step. In contrast, the motif-centric approach uses kinase to recognize specific substrates and transfer the phosphate group to back-phosphorylated peptides. Endogenous phosphopeptides, especially low-abundance tyrosine phosphopeptides, are not removed before IMAC enrichment. Therefore, only a few tens of micrograms of material was required for our isobaric motif-centric approach.

Recently, a novel antibody-based method, called PTMScan Direct, was developed for the identification and quantitation of peptides derived from proteins that are critical signaling nodes of various pathways (Stokes et al., 2012). However, the coverage of the IAPs approach is still limited by the availability and quality of antibodies. In contrast, recombinant active kinases are much more readily available than antibodies. In our previous study (Sugiyama et al., 2019), we were able to identify a total of 175,574 potential direct kinase substrates by using 385 active kinases (354 wild-type protein kinases, 21 mutants, and 10 lipid kinases). Based on this kinase substrate library, it is easily possible to select multiple kinases for targeting pathway analysis.

The multiplexing nature of isobaric labeling is particularly useful to achieve greater sensitivity with limited individual sample amounts, as in the case of tyrosine phosphopeptides. Chua et al. (2020) and Fang et al. (2020) used samples with and without PV (tyrosine phosphatase inhibitor)-treated cells as a boosting channel to increase the relative abundance of tyrosine phosphorylation sites. Although approximately 2,300 (Chua et al., 2020) and 835 pY phosphopeptides (Fang et al., 2020) were detected, the required amount of starting material is at the milligram level. In addition, the use of antibodies for pY phosphopeptides enrichment is also necessary for their approach. Here, in contrast, we generate higher purity tyrosine phosphopeptides via *in vitro* kinase reactions (motif-centric). By using motif-centric peptides, we could detect up to 7,129 (SRC targeting) and 7,280 (EGFR targeting) tyrosine phosphopeptides without the need for immunoprecipitation, using only a few tens of μg of starting materials.

Conclusions

The isobaric motif-centric strategy presented here can be used to enhance the sensitivity of specific kinase downstream signaling analysis, especially for tyrosine phosphopeptides. It provides a simple yet highly effective quantitative phosphoproteomic workflow suitable for multiplexed analysis of relatively small biological or clinical samples (less than milligram size), including cells or tissues. This approach enables the quantitation of both fold-change and stoichiometry among thousands of phosphopeptides generated by specific kinases in signaling pathways. The use of multiple kinases for motif targeting analysis effectively increases the phosphoproteome coverage. Overall, we anticipate this strategy should find broad biomedical applications for targeting kinase/pathway analysis where limited amounts of starting cells or tissues are available.

Limitations of the study

Although the performance of isobaric labeling-based quantitative approaches is affected by co-selected precursor ions, the use of SPS-MS^3 can minimize this effect and improve the detection sensitivity by using co-fragmented multiple ($\leq$10) MS^2 fragment ions with higher intensity (McAlister et al., 2014). Moreover, the newly available Real Time Search-MS^3 method (RTS-MS^3) (Erickson et al., 2019) or ion mobility technique (Bekker-Jensen et al., 2020; Hebert et al., 2018; Ogata and Ishihama, 2020) provides a solution for precise and accurate quantitation without sacrificing proteome coverage. Recently, the use of spiked TMT labeling peptides with boosting or carrier samples has decreased the TMT reporter ions dynamic range and decreased the quantitation accuracy (Tsai et al., 2020). Cheung et al. (2021) and Stopfer et al. (2021a) also demonstrated that an increase in carrier proteome level requires a concomitant increase in the number of ions sampled to maintain quantitative accuracy. Therefore, it will be important to optimize the spiking amount of carrier/boosting and the TMT channel design for the isobaric motif-centric strategy. As shown in Figure S7, the spiking amount of carrier/boosting affected the number of quantifiable pY sites (boost ratio <100). In addition, optimization of the MS parameters to improve the ion sampling by adjusting the ion injection time and AGC will also be beneficial to improve the quantitation performance (Cheung et al., 2021; Tsai et al., 2020). To overcome the challenge presented by the large quantitation dynamic range, it may be useful to integrate the motif-centric approach with isotope-based SureQuant (Stopfer et al., 2021b) or Internal Standard Triggered-Parallel Reaction Monitoring (Gallien et al., 2015) quantitation.

For the phosphorylation stoichiometry, it was assumed that the kinase reaction efficiency was 100%. Similar to our previous motif-targeting approach for measuring phosphorylation stoichiometry (Tsai et al., 2015), we used a high concentration of ATP (1 mM) and a long incubation time (overnight) to maximize the efficiency of the reaction. However, it is not always possible to achieve 100% kinase reaction efficiency for all sites. If the reaction efficiency is not 100%, then the measured stoichiometry will be overestimated.

Although we used a single kinase to produce the motif-centric peptides for each TMT set, it is possible to pool multiple kinase products to increase the throughput of this analysis. However, before applying this method to real samples, it is necessary to optimize how many and what kinases can be combined to avoid excessive increase in sample complexity and dynamic range. Otherwise, the quantification of peptides with low amounts would be problematic.

STAR★METHODS

Detailed methods are provided in the online version of this paper and include the following:

- KEY RESOURCES TABLE
- RESOURCE AVAILABILITY
  - Lead contact
  - Materials availability
  - Data and code availability
- EXPERIMENTAL MODEL AND SUBJECT DETAILS
- METHOD DETAILS
  - Tryptic peptides from HeLa cell lysate
  - *In vitro* kinase reactions
  - TMT labeling for digested peptides
  - IMAC
  - LC-MS/MS analysis
- QUANTIFICATION AND STATISTICAL ANALYSIS

SUPPLEMENTAL INFORMATION

Supplemental information can be found online at https://doi.org/10.1016/j.crmeth.2021.100138.

ACKNOWLEDGMENTS

We would like to thank all lab members for fruitful discussions. C.-F.T. was supported by a JSPS Grant-in-Aid for a postdoctoral fellowship for overseas researchers (15F15343). This work was supported by the JST Strategic Basic Research Program, CREST (grant No. 18070870) to Y.I., and by a JSPS Grants-in-Aid for Scientific Research (No. 17H03605, No. 21H02459 to Y.I., No. 20K21478 to Y.I. and K.O., and No. 20H04845, No. 21H02466 to N.S.)

AUTHOR CONTRIBUTIONS

Conceptualization, C.F.T. and Y.I.; Methodology, C.F.T. and Y.I.; Investigation, C.F.T., K.O. and N. S.; Writing – Original Draft, C.F.T.; Writing –Review & Editing, C.F.T. and Y.I; Funding Acquisition, Y.I.; Supervision, Y.I.

DECLARATION OF INTERESTS

The authors declare no competing interests.

Received: July 1, 2021
Revised: October 8, 2021
Accepted: December 13, 2021
Published: January 14, 2022

REFERENCES

Abe, Y., Nagano, M., Tada, A., Adachi, J., and Tomonaga, T. (2017). Deep phosphotyrosine proteomics by optimization of phosphotyrosine enrichment and MS/MS parameters. J. Proteome Res. *16*, 1077–1086.

Bekker-Jensen, D.B., Martinez-Val, A., Steigerwald, S., Ruther, P., Fort, K.L., Arrey, T.N., Harder, A., Makarov, A., and Olsen, J.V. (2020). A compact quadrupole-orbitrap mass spectrometer with FAIMS interface improves proteome coverage in short LC gradients. Mol. Cell. Proteomics *19*, 716–729.

Bian, Y., Li, L., Dong, M., Liu, X., Kaneko, T., Cheng, K., Liu, H., Voss, C., Cao, X., Wang, Y., et al. (2016). Ultra-deep tyrosine phosphoproteomics enabled by a phosphotyrosine superbinder. Nat. Chem. Biol. *12*, 959–966.

Bloom, J., and Cross, F.R. (2007). Multiple levels of cyclin specificity in cell-cycle control. Nat. Rev. Mol. Cell Biol. *8*, 149–160.

Budnik, B., Levy, E., Harmange, G., and Slavov, N. (2018). SCoPE-MS: mass spectrometry of single mammalian cells quantifies proteome heterogeneity during cell differentiation. Genome Biol. *19*, 161.

Cheung, T.K., Lee, C.Y., Bayer, F.P., McCoy, A., Kuster, B., and Rose, C.M. (2021). Defining the carrier proteome limit for single-cell proteomics. Nat. Methods *18*, 76–83.

Chon, H.J., Bae, K.J., Lee, Y., and Kim, J. (2015). The casein kinase 2 inhibitor, CX-4945, as an anti-cancer drug in treatment of human hematological malignancies. Front. Pharmacol. *6*, 70.

Chua, X.Y., Mensah, T., Aballo, T., Mackintosh, S.G., Edmondson, R.D., and Salomon, A.R. (2020). Tandem mass tag approach utilizing pervanadate BOOST channels delivers deeper quantitative characterization of the tyrosine phosphoproteome. Mol. Cell. Proteomics *19*, 730–743.

Cohen, P. (2002). The origins of protein phosphorylation. Nat. Cell Biol. *4*, E127–E130.

Cox, J., and Mann, M. (2008). MaxQuant enables high peptide identification rates, individualized p.p.b.-range mass accuracies and proteome-wide protein quantification. Nat. Biotechnol. *26*, 1367–1372.

Dong, M., Bian, Y., Wang, Y., Dong, J., Yao, Y., Deng, Z., Qin, H., Zou, H., and Ye, M. (2017). Sensitive, robust, and cost-effective approach for tyrosine phosphoproteome analysis. Anal. Chem. *89*, 9307–9314.

Dou, M., Clair, G., Tsai, C.F., Xu, K., Chrisler, W.B., Sontag, R.L., Zhao, R., Moore, R.J., Liu, T., Pasa-Tolic, L., et al. (2019). High-throughput single cell proteomics enabled by multiplex isobaric labeling in a nanodroplet sample preparation platform. Anal. Chem. *91*, 13119–13127.

Erickson, B.K., Mintseris, J., Schweppe, D.K., Navarrete-Perea, J., Erickson, A.R., Nusinow, D.P., Paulo, J.A., and Gygi, S.P. (2019). Active instrument engagement combined with a real-time database search for improved performance of sample multiplexing workflows. J. Proteome Res. *18*, 1299–1306.

Fang, B., Izumi, V., Rix, L.L.R., Welsh, E., Pike, I., Reuther, G.W., Haura, E.B., Rix, U., and Koomen, J.M. (2020). Lowering sample requirements to study tyrosine kinase signaling using phosphoproteomics with the TMT calibrator approach. Proteomics *20*, e2000116.

Gallien, S., Kim, S.Y., and Domon, B. (2015). Large-scale targeted proteomics using internal standard triggered-parallel reaction monitoring (IS-PRM). Mol. Cell. Proteomics *14*, 1630–1644.

Hebert, A.S., Prasad, S., Belford, M.W., Bailey, D.J., McAlister, G.C., Abbatiello, S.E., Huguet, R., Wouters, E.R., Dunyach, J.J., Brademan, D.R., et al. (2018). Comprehensive single-shot proteomics with FAIMS on a hybrid Orbitrap mass spectrometer. Anal. Chem. *90*, 9529–9537.

Hogrebe, A., von Stechow, L., Bekker-Jensen, D.B., Weinert, B.T., Kelstrup, C.D., and Olsen, J.V. (2018). Benchmarking common quantification strategies for large-scale phosphoproteomics. Nat. Commun. *9*, 1045.

Hornbeck, P.V., Zhang, B., Murray, B., Kornhauser, J.M., Latham, V., and Skrzypek, E. (2015). PhosphoSitePlus, 2014: mutations, PTMs and recalibrations. Nucleic Acids Res. *43*, D512–D520.

Huang da, W., Sherman, B.T., and Lempicki, R.A. (2009). Systematic and integrative analysis of large gene lists using DAVID bioinformatics resources. Nat. Protoc. *4*, 44–57.

Hughes, C.S., Zhu, C., Spicer, V., Krokhin, O.V., and Morin, G.B. (2017). Evaluating the characteristics of reporter ion signal acquired in the Orbitrap analyzer for isobaric mass tag proteome quantification experiments. J. Proteome Res. *16*, 1831–1838.

Humphrey, S.J., Azimifar, S.B., and Mann, M. (2015). High-throughput phosphoproteomics reveals in vivo insulin signaling dynamics. Nat. Biotechnol. *33*, 990–995.

Hunter, T. (2000). Signaling–2000 and beyond. Cell *100*, 113–127.

Hunter, T. (2009). Tyrosine phosphorylation: thirty years and counting. Curr. Opin. Cell Biol. *21*, 140–146.

Hunter, T., and Sefton, B.M. (1980). Transforming gene product of Rous sarcoma virus phosphorylates tyrosine. Proc. Natl. Acad. Sci. U S A *77*, 1311–1315.

Imamura, H., Sugiyama, N., Wakabayashi, M., and Ishihama, Y. (2014). Large-scale identification of phosphorylation sites for profiling protein kinase selectivity. J. Proteome Res. *13*, 3410–3419.

Imamura, H., Wagih, O., Niinae, T., Sugiyama, N., Beltrao, P., and Ishihama, Y. (2017). Identifications of putative PKA substrates with quantitative phosphoproteomics and primary-sequence-based scoring. J. Proteome Res. *16*, 1825–1830.

Iwasaki, M., Miwa, S., Ikegami, T., Tomita, M., Tanaka, N., and Ishihama, Y. (2010). One-dimensional capillary liquid chromatographic separation coupled with tandem mass spectrometry unveils the Escherichia coli proteome on a microarray scale. Anal. Chem. *82*, 2616–2620.

Li, X., Cox, J.T., Huang, W., Kane, M., Tang, K., and Bieberich, C.J. (2016). Quantifying kinase-specific phosphorylation stoichiometry using stable isotope labeling in a reverse in-gel kinase assay. Anal. Chem. *88*, 11468–11475.

Masuda, T., Tomita, M., and Ishihama, Y. (2008). Phase transfer surfactant-aided trypsin digestion for membrane proteome analysis. J. Proteome Res. *7*, 731–740.

McAlister, G.C., Nusinow, D.P., Jedrychowski, M.P., Wuhr, M., Huttlin, E.L., Erickson, B.K., Rad, R., Haas, W., and Gygi, S.P. (2014). MultiNotch MS3 enables accurate, sensitive, and multiplexed detection of differential expression across cancer cell line proteomes. Anal. Chem. *86*, 7150–7158.

Mertins, P., Tang, L.C., Krug, K., Clark, D.J., Gritsenko, M.A., Chen, L., Clauser, K.R., Clauss, T.R., Shah, P., Gillette, M.A., et al. (2018). Reproducible workflow for multiplexed deep-scale proteome and phosphoproteome analysis of tumor tissues by liquid chromatography-mass spectrometry. Nat. Protoc. *13*, 1632–1661.

Moriya, Y., Kawano, S., Okuda, S., Watanabe, Y., Matsumoto, M., Takami, T., Kobayashi, D., Yamanouchi, Y., Araki, N., Yoshizawa, A.C., et al. (2019). The jPOST environment: an integrated proteomics data repository and database. Nucleic Acids Res. *47*, D1218–D1224.

Mundina-Weilenmann, C., Chang, C.F., Gutierrez, L.M., and Hosey, M.M. (1991). Demonstration of the phosphorylation of dihydropyridine-sensitive calcium channels in chick skeletal muscle and the resultant activation of the channels after reconstitution. J. Biol. Chem. *266*, 4067–4073.

Needham, E.J., Parker, B.L., Burykin, T., James, D.E., and Humphrey, S.J. (2019). Illuminating the dark phosphoproteome. Sci. Signal. *12*, eaau8645.

O'Shea, J.P., Chou, M.F., Quader, S.A., Ryan, J.K., Church, G.M., and Schwartz, D. (2013). pLogo: a probabilistic approach to visualizing sequence motifs. Nat. Methods *10*, 1211–1212.

Ogata, K., and Ishihama, Y. (2020). Extending the separation space with trapped ion mobility spectrometry improves the accuracy of isobaric tag-based quantitation in proteomic LC/MS/MS. Anal. Chem. *92*, 8037–8040.

Ogata, K., Tsai, C.F., and Ishihama, Y. (2021). Nanoscale solid-phase isobaric labeling for multiplexed quantitative phosphoproteomics. J. Proteome Res. *20*, 4193–4202.

Possemato, A.P., Paulo, J.A., Mulhern, D., Guo, A., Gygi, S.P., and Beausoleil, S.A. (2017). Multiplexed phosphoproteomic profiling using titanium dioxide and immunoaffinity enrichments reveals complementary phosphorylation events. J. Proteome Res. *16*, 1506–1514.

Rappsilber, J., Mann, M., and Ishihama, Y. (2007). Protocol for micro-purification, enrichment, pre-fractionation and storage of peptides for proteomics using StageTips. Nat. Protoc. *2*, 1896–1906.

Russo, G.L., Vandenberg, M.T., Yu, I.J., Bae, Y.S., Franza, B.R., Jr., and Marshak, D.R. (1992). Casein kinase II phosphorylates p34cdc2 kinase in G1 phase of the HeLa cell division cycle. J. Biol. Chem. *267*, 20317–20325.

Sharma, K., D'Souza, R.C., Tyanova, S., Schaab, C., Wisniewski, J.R., Cox, J., and Mann, M. (2014). Ultradeep human phosphoproteome reveals a distinct regulatory nature of Tyr and Ser/Thr-based signaling. Cell Rep. *8*, 1583–1594.

Songyang, Z., Lu, K.P., Kwon, Y.T., Tsai, L.H., Filhol, O., Cochet, C., Brickey, D.A., Soderling, T.R., Bartleson, C., Graves, D.J., et al. (1996). A structural basis for substrate specificities of protein Ser/Thr kinases: primary sequence preference of casein kinases I and II, NIMA, phosphorylase kinase, calmodulin-dependent kinase II, CDK5, and Erk1. Mol. Cell. Biol. *16*, 6486–6493.

Stokes, M.P., Farnsworth, C.L., Moritz, A., Silva, J.C., Jia, X., Lee, K.A., Guo, A., Polakiewicz, R.D., and Comb, M.J. (2012). PTMScan direct: identification and quantification of peptides from critical signaling proteins by immunoaffinity enrichment coupled with LC-MS/MS. Mol. Cell. Proteomics *11*, 187–201.

Stokes, M.P., Farnsworth, C.L., Gu, H., Jia, X., Worsfold, C.R., Yang, V., Ren, J.M., Lee, K.A., and Silva, J.C. (2015). Complementary PTM profiling of drug response in human gastric carcinoma by immunoaffinity and IMAC methods with total proteome analysis. Proteomes *3*, 160–183.

Stopfer, L.E., Conage-Pough, J.E., and White, F.M. (2021a). Quantitative consequences of protein carriers in immunopeptidomics and tyrosine phosphorylation MS(2) analyses. Mol. Cell. Proteomics *20*, 100104.

Stopfer, L.E., Flower, C.T., Gajadhar, A.S., Patel, B., Gallien, S., Lopez-Ferrer, D., and White, F.M. (2021b). High-density, targeted monitoring of tyrosine phosphorylation reveals activated signaling networks in human tumors. Cancer Res. *81*, 2495–2509.

Sugiyama, N., Imamura, H., and Ishihama, Y. (2019). Large-scale discovery of substrates of the human kinome. Sci. Rep. *9*, 10503.

Szklarczyk, D., Gable, A.L., Lyon, D., Junge, A., Wyder, S., Huerta-Cepas, J., Simonovic, M., Doncheva, N.T., Morris, J.H., Bork, P., et al. (2019). STRING v11: protein-protein association networks with increased coverage, supporting functional discovery in genome-wide experimental datasets. Nucleic Acids Res. *47*, D607–D613.

Thompson, A., Schafer, J., Kuhn, K., Kienle, S., Schwarz, J., Schmidt, G., Neumann, T., Johnstone, R., Mohammed, A.K., and Hamon, C. (2003). Tandem mass tags: a novel quantification strategy for comparative analysis of complex protein mixtures by MS/MS. Anal. Chem. *75*, 1895–1904.

Tsai, C.F., Hsu, C.C., Hung, J.N., Wang, Y.T., Choong, W.K., Zeng, M.Y., Lin, P.Y., Hong, R.W., Sung, T.Y., and Chen, Y.J. (2014). Sequential phosphoproteomic enrichment through complementary metal-directed immobilized metal ion affinity chromatography. Anal. Chem. *86*, 685–693.

Tsai, C.F., Wang, Y.T., Yen, H.Y., Tsou, C.C., Ku, W.C., Lin, P.Y., Chen, H.Y., Nesvizhskii, A.I., Ishihama, Y., and Chen, Y.J. (2015). Large-scale determination of absolute phosphorylation stoichiometries in human cells by motif-targeting quantitative proteomics. Nat. Commun. *6*, 6622.

Tsai, C.F., Zhao, R., Williams, S.M., Moore, R.J., Schultz, K., Chrisler, W.B., Pasa-Tolic, L., Rodland, K.D., Smith, R.D., Shi, T., et al. (2020). An improved boosting to amplify signal with isobaric labeling (iBASIL) strategy for precise quantitative single-cell proteomics. Mol. Cell. Proteomics *19*, 828–838.

Tyanova, S., Temu, T., and Cox, J. (2016a). The MaxQuant computational platform for mass spectrometry-based shotgun proteomics. Nat. Protoc. *11*, 2301–2319.

Tyanova, S., Temu, T., Sinitcyn, P., Carlson, A., Hein, M.Y., Geiger, T., Mann, M., and Cox, J. (2016b). The Perseus computational platform for comprehensive analysis of (prote)omics data. Nat. Methods *13*, 731–740.

Villen, J., Beausoleil, S.A., Gerber, S.A., and Gygi, S.P. (2007). Large-scale phosphorylation analysis of mouse liver. Proc. Natl. Acad. Sci. U S A *104*, 1488–1493.

Wang, Y.T., Pan, S.H., Tsai, C.F., Kuo, T.C., Hsu, Y.L., Yen, H.Y., Choong, W.K., Wu, H.Y., Liao, Y.C., Hong, T.M., et al. (2017). Phosphoproteomics reveals HMGA1, a CK2 substrate, as a drug-resistant target in non-small cell lung cancer. Sci. Rep. *7*, 44021.

Xue, L., Wang, W.H., Iliuk, A., Hu, L., Galan, J.A., Yu, S., Hans, M., Geahlen, R.L., and Tao, W.A. (2012). Sensitive kinase assay linked with phosphoproteomics for identifying direct kinase substrates. Proc. Natl. Acad. Sci. U S A *109*, 5615–5620.

Xue, L., Geahlen, R.L., and Tao, W.A. (2013). Identification of direct tyrosine kinase substrates based on protein kinase assay-linked phosphoproteomics. Mol. Cell. Proteomics *12*, 2969–2980.

Yi, L., Tsai, C.F., Dirice, E., Swensen, A.C., Chen, J., Shi, T., Gritsenko, M.A., Chu, R.K., Piehowski, P.D., Smith, R.D., Rodland, K.D., Atkinson, M.A., Mathews, C.E., Kulkarni, R.N., Liu, T., and Qian, W.J. (2019). Boosting to amplify signal with isobaric labeling (BASIL) strategy for comprehensive quantitative phosphoproteomic characterization of small populations of cells. Anal. Chem. *75*, 5794–5801.

CellPress
OPEN ACCESS

STAR★METHODS

KEY RESOURCES TABLE

REAGENT or RESOURCE	SOURCE	IDENTIFIER
Chemicals, peptides, and recombinant proteins		
Triethylammonium bicarbonate	Sigma	Catalog: T7408
Phosphatase Inhibitor Cocktail 2	Sigma	Catalog: P5726
Phosphatase Inhibitor Cocktail 3	Sigma	Catalog: P0044
BCA Protein Assay Kit	Thermo Scientific Pierce	Catalog: 23225
Sodium deoxycholate	FUJIFILM Wako	Catalog: 190-08313
Sodium lauroyl sarcosinate	FUJIFILM Wako	Catalog: 198-14745
Iron-(III) chloride	FUJIFILM Wako	Catalog: 091-00872
Dithiothreitol	Thermo Scientific	Catalog: 20291
Iodoacetamide	Thermo Scientific	Catalog: A3221
Lysyl endopeptidase	FUJIFILM Wako	Catalog: 129-02541
Sequencing-grade modified trypsin	Promega	Catalog: V517
Ni-NTA silica resins	QIAGEN	Catalog: 31314
Empore SDB-XC membrane disks	CDS	Catalog: 13-110-020
Titanium dioxide (10 μm)	GL Sciences	Catalog: 5020-75010
CK2α2/β (CSNK2A2/B)	Carna Biosciences	Catalog:05-185
PKACα(PRKACA)	Carna Biosciences	Catalog:01-127
ERK2 (MAPK1)	Carna Biosciences	Catalog:04-143
EGFR (ERBB1)	Carna Biosciences	Catalog:08-115
SRC	Carna Biosciences	Catalog:08-173
JNK1(MAPK8)	Carna Biosciences	Catalog:04-163
CDK1 (CDC2/CycB1)	Carna Biosciences	Catalog:04-102
p38α(MAPK14)	Carna Biosciences	Catalog:04-152
TMTsixplex™	Thermo Scientific	Catalog:90061
Deposited data		
Zenodo	https://zenodo.org/	https://doi.org/10.5281/zenodo.5750874
Experimental models: Cell lines		
HeLa S3	ATCC	Catalog: CCL-2.2
Software and algorithms		
MaxQuant	PMID: 27809316	https://www.maxquant.org/
Perseus	PMID: 27348712	https://maxquant.net/perseus/

RESOURCE AVAILABILITY

Lead contact

Further information and requests for resources and reagents should be directed to and will be fulfilled by the lead contact, Yasushi Ishihama (yishiham@pharm.kyoto-u.ac.jp).

Materials availability

This study did not generate new unique reagents.

Data and code availability

- Data described in this paper have been deposited at https://zenodo.org and are publicly available as of the date of publication. DOIs are listed in the key resources table.
- This paper does not report original code.
- Any additional information required to reanalyze the data reported in this paper is available from the lead contact upon request

EXPERIMENTAL MODEL AND SUBJECT DETAILS

HeLa S3 cells were cultured in DMEM containing 10% fetal bovine serum and 100 μg/mL kanamycin. For isobaric acidophilic motif-centric phosphoproteomes, cells were not stimulated (mock) or were stimulated with 10 μM CK2 inhibitor (CX-4945) for 30 min. For isobaric basophilic motif-centric phosphoproteomes, cells were not stimulated (mock) or were stimulated with 10 μM PKA activator (forskolin) for 30 min. For isobaric tyrosine and multiple motif-centric phosphoproteome, cells were treated with 10 μM EGF, 10 μM EGF/10 μM afatinib, and 500 μM PV (pH 10 with 0.14% H_2O_2), respectively, for 30 min before harvesting. Two biological replicates were performed.

METHOD DETAILS

Tryptic peptides from HeLa cell lysate

Cells were washed three times with ice-cold phosphate-buffered saline (phosphate-buffered saline, 0.01 M sodium phosphate, 0.14 M NaCl, pH 7.4) and then lysed in lysis buffer containing 12 mM sodium deoxycholate, 12 mM sodium lauroyl sarcosinate in 100 mM triethylammonium bicarbonate. Protein concentration was determined by means of BCA protein assay. The lysates were digested based on the reported phase-transfer surfactants protocol (Masuda et al., 2008). The digested peptides were desalted on SDB-XC StageTips (Rappsilber et al., 2007).

In vitro kinase reactions

For acidophilic, Pro-directed and tyrosine kinase reactions, the tryptic peptides were dissolved in 40 mM Tris-HCl (pH 7.5) and incubated with each kinase (0.2 μg CK2, ERK2, JNK1, p38α, CDK1 or SRC) at 37°C overnight for *in vitro* kinase reactions in the presence of 1 mM ATP and 20 mM $MgCl_2$. For the EGFR kinase reactions, tryptic peptides were firstly passed through SCX StageTips (Rappsilber et al., 2007) to remove afatinib. Eluted peptides were further desalted on SDB-XC StageTips. Then, the desalted peptides were dissolved in 40 mM Tris-HCl (pH 7.5) and incubated with EGFR (0.2 μg) for *in vitro* kinase reactions in the presence of 1 mM ATP and 4 mM $MnCl_2$ at 37°C overnight. For basophilic kinases such as PKA, the lysates were loaded onto a 10-kDa ultrafiltration device (Amicon Ultra, Millipore). The device was centrifuged at 14,000 *g* to remove the detergents. Subsequently, the original lysis buffer was replaced with 40 mM Tris-HCl (pH 7.5) followed by centrifugation. Then, the proteins were incubated with 0.2 μg PKA for *in vitro* kinase reactions in the presence of 1 mM ATP and 20 mM $MgCl_2$ at 37°C overnight. After the kinase reactions, the proteins were reduced with 10 mM DTT for 30 min at 37°C and alkylated with 50 mM iodoacetamide in the dark for 30 min at 37°C. The resulting samples were digested by Lys-C (1:100, w/w) at 37°C for 3 h followed by trypsin (1:50, w/w) overnight at 37°C. All the peptides were desalted on SDB-XC StageTips.

TMT labeling for digested peptides

The desalted peptides were dissolved in 200 mM HEPES (pH 8.5). Then, the resuspended digested peptides were mixed with TMT reagent dissolved in 100% ACN for 1 h. The labeling reaction was stopped by adding 5% hydroxylamine for 15 min, followed by acidification with TFA. All the peptides labeled with each multiplexed TMT reagent were mixed into the same tube and the mixture was diluted to decrease the concentration of ACN to less than 5%. The TMT-labeled peptides were desalted on SDB-XC StageTips. The information on the peptide amount in each TMT channel for all experiments is shown in Table S3. Note that the peptide amount for each TMT channel was quantified by means of nanoLC-UV at 210 nm using a Thermo Ultimate 3000 RSLCnano system (Germering), an MU701 UV detector (GL Sciences), and a C18 analytical column (150 mm length × 100 μm ID) packed with Reprosil-Pur 120 C18-AQ material (3 μm, Dr. Maisch).

IMAC

The procedure for phosphopeptides purification with an Fe^{3+}-IMAC tip was as described previously (Tsai et al., 2014, 2015) with minor modifications. In brief, a buffer consisting of 50 mM EDTA in 1 M NaCl was used for removing Ni^{2+} ions. Then, the metal-free NTA was activated by loading 100 mM $FeCl_3$ into the IMAC tip. The Fe^{3+}-IMAC tip was equilibrated with 0.5% (v/v) acetic acid at pH 3.0 before sample loading. Tryptic peptides from HeLa lysates were reconstituted in 0.5% (v/v) acetic acid and loaded onto the IMAC tip. After successive washing steps with 1% (v/v) TFA in 80% ACN and 0.5% (v/v) acetic acid, the IMAC tip was coupled to an activated SDB-XC StageTip and the bound phosphopeptides were eluted onto the SDB-XC StageTip with 200 mM $NH_4H_2PO_4$ buffer. Then, the eluted phosphopeptides were desalted with SDB-XC StageTip.

LC-MS/MS analysis

NanoLC-MS/MS analyses were performed on an Orbitrap Fusion Lumos Tribrid mass spectrometer (Thermo Scientific), which was connected to the Thermo Ultimate 3000 RSLCnano system and an HTC-PAL autosampler (CTC Analytics). Peptide mixtures were loaded onto and separated on self-pulled needle columns (150 mm length × 100 μm inner diameter) packed with Reprosil-Pur 120 C18-AQ material (3 μm) or a 2-m-long C18 monolithic silica capillary column (Iwasaki et al., 2010). The mobile phases consisted of (A) 0.5% acetic acid and (B) 0.5% acetic acid and 80% acetonitrile. Peptides were separated through a gradient from 17.5 % to 45% buffer B at a flow rate of 500 nL/min. Full-scan spectra were acquired at a target value of 4×10^5 with a resolution of 60,000. Data

were acquired in a data-dependent acquisition mode using the top-speed method (3 s). The peptides were isolated using a quadrupole system (the isolation window was 0.7). The MS^2 analysis was performed in the ion trap using collision-induced dissociation fragmentation with a collision energy of 35 at a target value of 1×10^4 with 100 ms maximum injection time. The MS^3 analysis was performed for each MS^2 scan acquired by using multiple MS^2 fragment ions isolated by an ion trap as precursors for the MS^3 analysis with a multinotch isolation waveform (McAlister et al., 2014). HCD fragmentation was used for MS^3 scan with an NCE of 65%, and the fragment ions were detected by the Orbitrap (resolution 15,000). The AGC target was 5×10^4 with a maximum ion injection time of 22 ms. The raw data sets have been deposited at the ProteomeXchange Consortium (http://proteomecentral.proteomexchange.org) via the jPOST partner repository (https://jpostdb.org) (Moriya et al., 2019) with the dataset identifier JPST001027 (PXD026996).

Data analyses

Database search. The raw MS/MS data were processed with MaxQuant (Cox and Mann, 2008; Tyanova et al., 2016a). Peptide search with full tryptic digestion and a maximum of two missed cleavages was performed against the SwissProt human database (20,102 entries). The mass tolerance for precursor and MS^3 ions was 4.5 ppm, whereas the tolerance for MS^2 ions was 0.5 Th. Acetylation (protein *N*-terminal), oxidation (M) and phospho (STY) were set as variable modifications and carbamidomethyl (C) was set as a fixed modification. The quantitation function of reporter ion MS^3 (6-plexed TMT) was turned on. The false discovery rate was set to 1% at the level of PSMs and proteins. A score cut-off of 40 was used for identified modified peptides.

QUANTIFICATION AND STATISTICAL ANALYSIS

The abundances of TMT were $\log_2$-transformed and further analyzed by Perseus (Tyanova et al., 2016b) for statistical evaluation such as principal component analyses and *t* tests. The PSP logo generator (Hornbeck et al., 2015) was used for sequence motif analysis. DAVID (Huang da et al., 2009) was used for gene ontology and pathway enrichment analysis. STRING v11 (Szklarczyk et al., 2019) was used for protein-protein interaction analysis. SigmaPlot (Systat Software), was used for preparing box plots.

Cell Reports Methods, Volume *2*

Supplemental information

Motif-centric phosphoproteomics to target kinase-mediated signaling pathways

Chia-Feng Tsai, Kosuke Ogata, Naoyuki Sugiyama, and Yasushi Ishihama

Supplemental Information

Motif-centric phosphoproteomics to target kinase-mediated signaling pathways

Chia-Feng Tsai[1,3,4], Kosuke Ogata[1,3], Naoyuki Sugiyama[1], Yasushi Ishihama[1,2]

[1]Graduate School of Pharmaceutical Sciences, Kyoto University, Kyoto 606-8501, Japan.

[2]Laboratory of Clinical and Analytical Chemistry, National Institute of Biomedical Innovation, Health and Nutrition, Ibaraki, Osaka, 567-0085, Japan.

[3]These authors contributed equally.

[4]Current address: Biological Sciences Division, Pacific Northwest National Laboratory, Richland, WA 99354, USA.

*Correspondence and lead contact: yishiham@pharm.kyoto-u.ac.jp

Tel: +81-75-753-4555

Fax: +81-75-753-4601

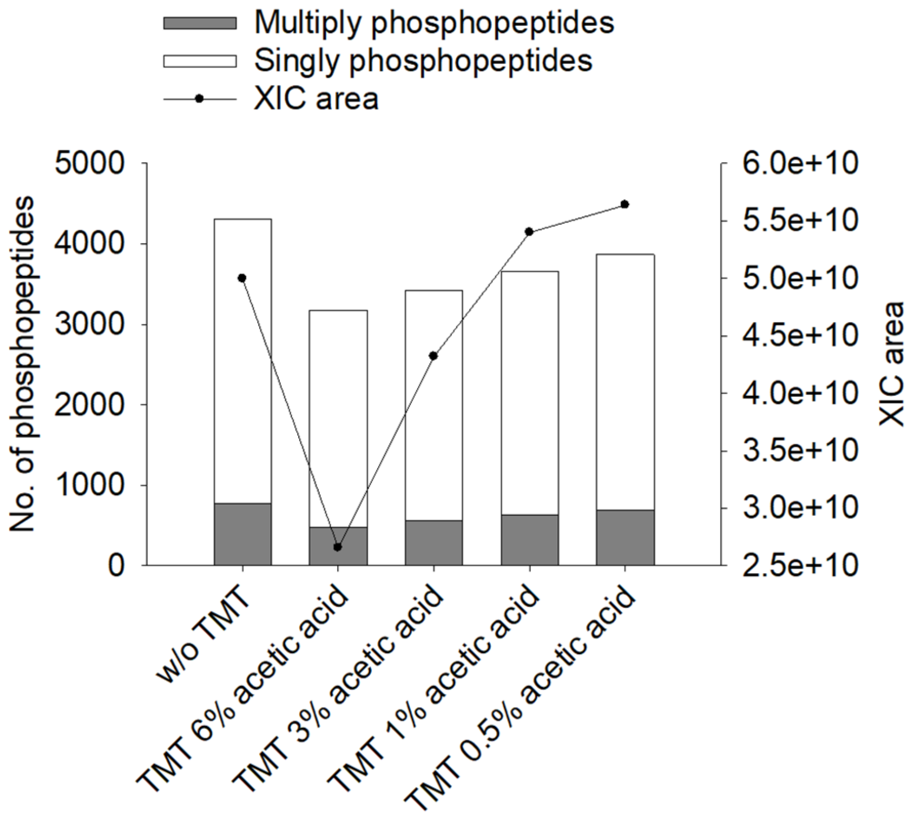

Figure S1. Effects of acetic acid concentration in the loading buffer on the enrichment of TMT-phosphopeptides by IMAC. TMT0 reagent was used for labeling 50 µg of HeLa tryptic peptides and IMAC enrichment followed by LC/MS/MS measurement with label free quantitation was performed for each sample (n=1). The optimized condition was used for phosphopeptide enrichment shown in Figure 1a.

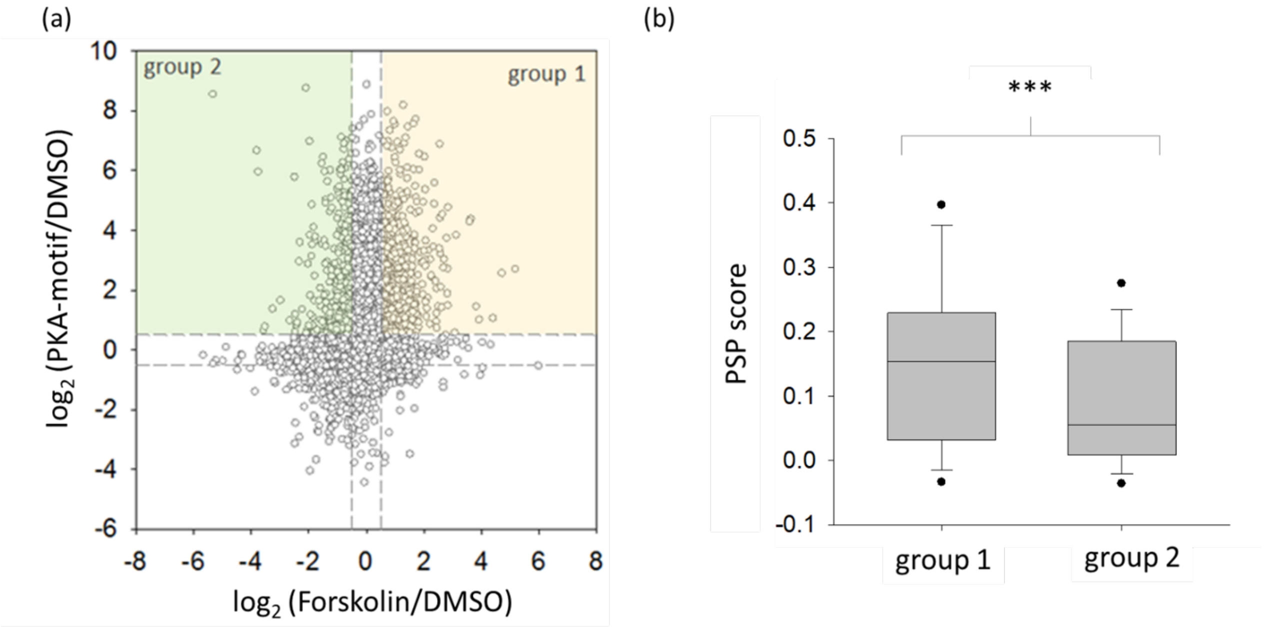

Figure S2. PKA motif-centric phosphoproteome analysis. Related to acidophilic CK2 phosphorylation shown in Figure 2, but *in vitro* phosphorylation was done at protein level in this case. (a) Signal intensity ratios of 620 phosphorylation sites phosphorylated *in vitro* by PKA increased after forskolin treatment (group 1), while 322 phosphorylation sites phosphorylated *in vitro* by PKA were inhibited by forskolin (group 2). (b) PSP score distribution of group 1 and group 2. *** $p<0.001$. Duplicate sample preparation and triplicate LC/MS/MS analyses were performed for each TMT set as shown in Table S3.

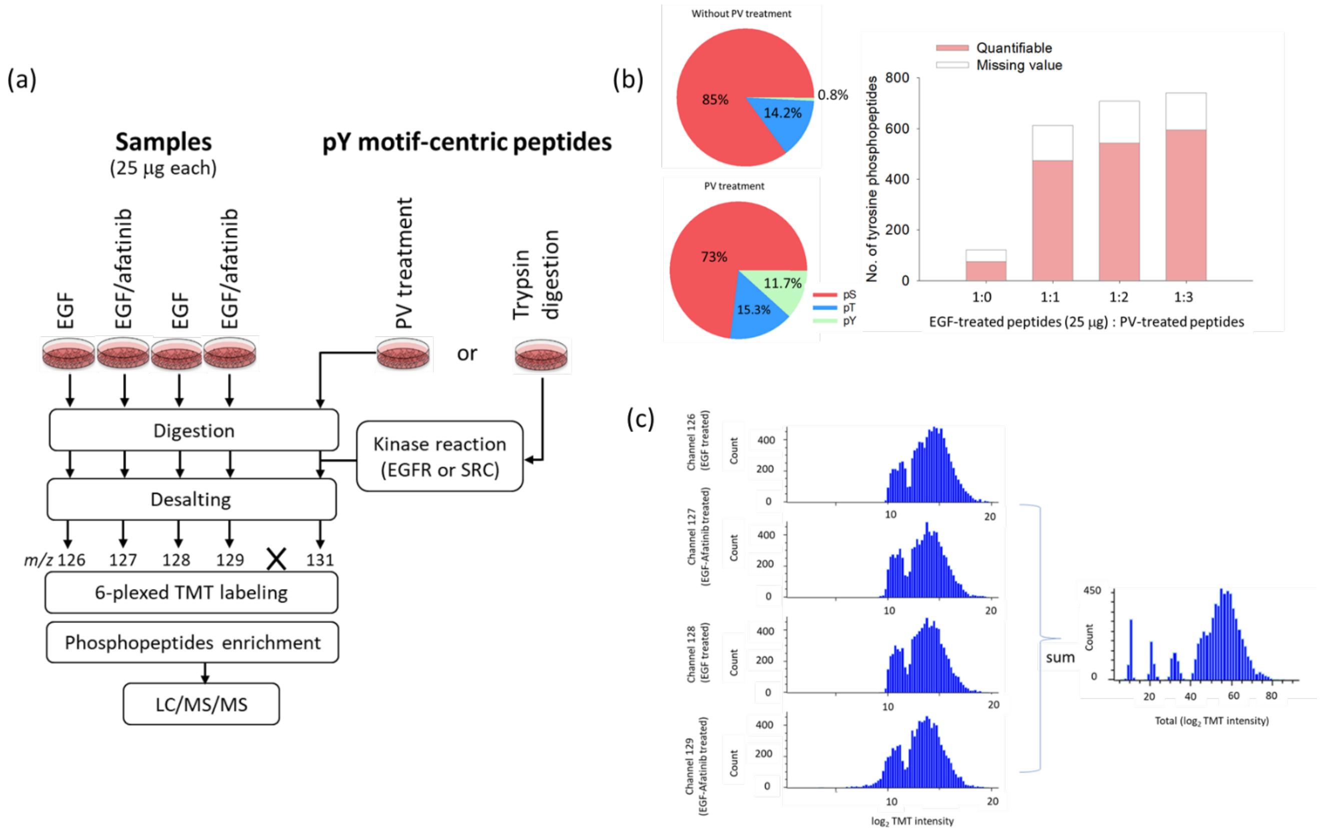

Figure S3. Experimental design for isobaric pY motif-centric phosphoproteomic approach. Related to Figure 3. (a) The pY-centric samples were generated by PV treatment or *in vitro* kinase reaction with EGFR or SRC. The tryptic peptides from study samples and pY motif-centric samples were labeled with multiplexed TMT tags, and were enriched by IMAC and analyzed by LC/MS/MS. (b) Effects of PV treatment on pY identification**.** Contents of pS, pT and pY sites identified from the IMAC-enriched tryptic peptides from PV-treated HeLa cells and untreated HeLa cells are shown in the pie charts (left). The numbers of quantifiable pY peptides from 25 μg of EGF-treated HeLa peptides, 25 μg of EGF/afatinib-treated HeLa peptides and different amounts of PV-treated peptides analyzed according to the workflow (a) are shown (right). A quantifiable pY peptide is defined as a pY peptide that contains the signals of TMT reporter ions in all TMT channels of the MS^3 spectrum. Otherwise, pY peptides are considered as missing values. (c) TMT signal distribution in each TMT reporter channel (left) and the merged signals from all channels except the motif-centric channel (right). Duplicate sample preparation for each TMT set was performed as shown in Table S3.

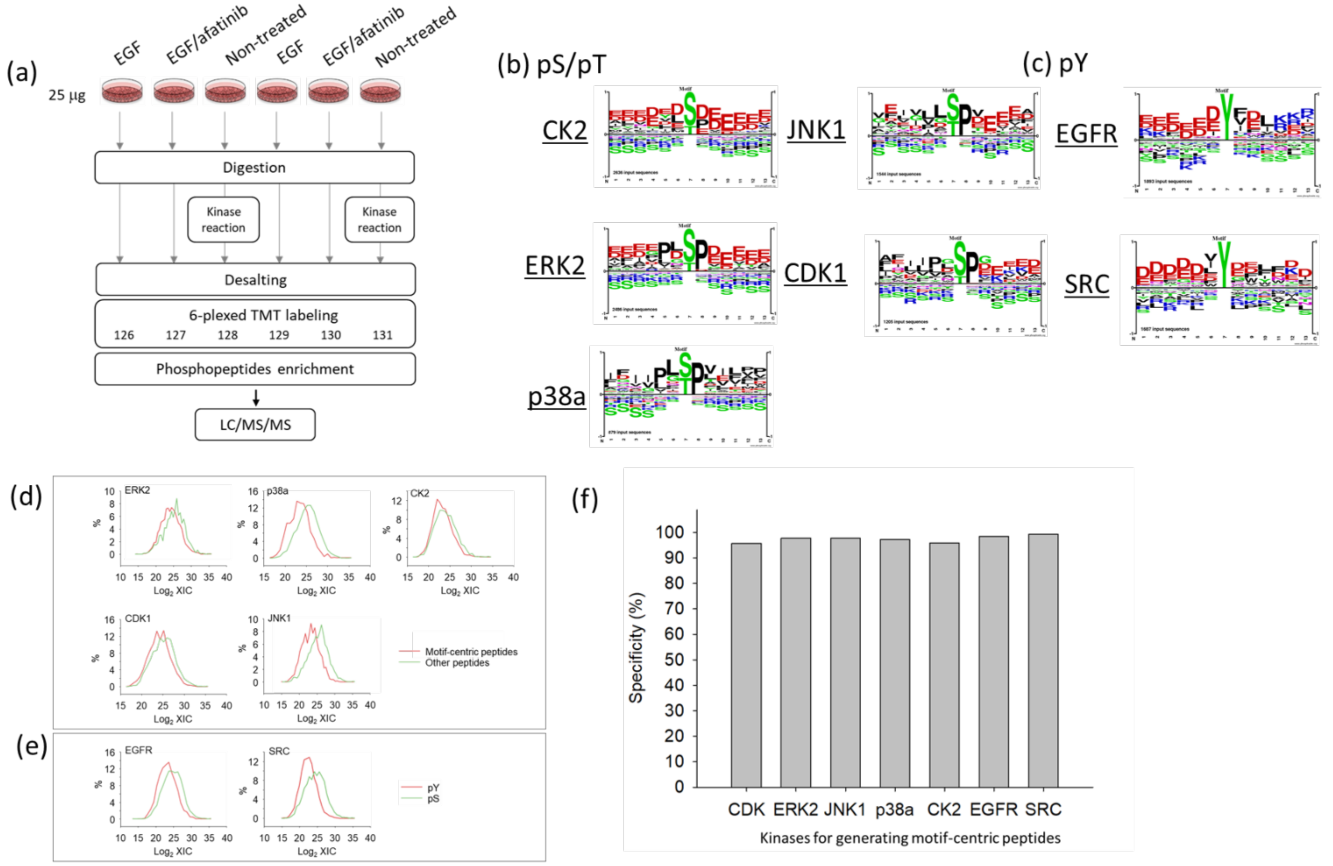

Figure S4. Isobaric motif-centric phosphoproteomics results with multiple kinases. Related to Figure 5. (a) Workflow of isobaric motif-centric phosphoproteomic approach with multiple kinases. Seven kinases, i.e., Pro-directed kinases ((ERK1 (MAPK3), JNK1(MAPK8), P38a (MAPK14) and CDK1)), tyrosine kinases (SRC and EGFR) and acidophilic kinase (CK2), were employed to generate motif-centric phosphopeptides. The tryptic peptides from study samples and motif-centric peptides were labeled with 6-plexed TMT tags. The TMT labeling phosphopeptides were enriched by IMAC and analyzed by LC/MS/MS. (b) Phosphorylation motif analysis for the motif-centric pS/pT sites. (c) Phosphorylation motif analysis for the motif-centric pY sites. (d) The distribution of XIC signals in MS1 for endogenous pS/pT peptides. (e) The distribution of XIC signals in MS1 for endogenous pY peptides. The XIC signals of the endogenous phosphopeptides in MS1 were calculated based on the TMT ratio between study samples and the motif-centric sample. (f) IMAC enrichment specificity for phosphopeptides based on peptide counts for each kinase experiment. Duplicate sample preparation and 2 or 3 replicate LC/MS/MS analyses were performed for each kinase as shown in Table S3.

Table S1. GO enrichment analysis of downregulated CK2-motif centric peptides (related to Figure 2d).

#Term ID	Term description	Observed gene count	Background gene count	False discovery rate
**Molecular function**				
GO:0005488	binding	168	11878	1.21E-11
GO:0005515	protein binding	112	6605	7.07E-09
GO:0005524	ATP binding	44	1462	7.07E-09
GO:0097159	organic cyclic compound binding	98	5382	7.07E-09
GO:1901363	heterocyclic compound binding	97	5305	7.07E-09
GO:0000166	nucleotide binding	53	2097	1.74E-08
GO:0016887	ATPase activity	22	392	1.74E-08
GO:0035639	purine ribonucleoside triphosphate binding	48	1794	1.78E-08
GO:0032555	purine ribonucleotide binding	48	1853	4.70E-08
GO:0008144	drug binding	45	1710	9.06E-08
GO:0036094	small molecule binding	56	2460	1.01E-07
GO:0043168	anion binding	59	2696	1.31E-07
GO:0017111	nucleoside-triphosphatase activity	28	778	2.68E-07
GO:0003676	nucleic acid binding	66	3332	5.02E-07
GO:0097367	carbohydrate derivative binding	50	2163	5.02E-07
GO:0051082	unfolded protein binding	10	106	6.28E-06
GO:0003677	DNA binding	51	2457	7.84E-06
GO:0043167	ion binding	92	6066	5.98E-05
GO:0042623	ATPase activity, coupled	14	320	0.00015
GO:0004386	helicase activity	9	147	0.00059
GO:0070182	DNA polymerase binding	4	15	0.0007
GO:0008026	ATP-dependent helicase activity	7	90	0.001
GO:0044877	protein-containing complex binding	24	968	0.001
GO:0019899	enzyme binding	41	2197	0.0014
GO:0004004	ATP-dependent RNA helicase activity	6	66	0.0015
GO:0140097	catalytic activity, acting on DNA	9	173	0.0016
GO:0008092	cytoskeletal protein binding	21	882	0.0043
GO:0008017	microtubule binding	10	253	0.005
GO:0016787	hydrolase activity	42	2448	0.0056
GO:0003824	catalytic activity	78	5592	0.0079
GO:0003697	single-stranded DNA binding	6	99	0.0094
GO:0008094	DNA-dependent ATPase activity	5	66	0.0102
GO:0015631	tubulin binding	11	344	0.0124
GO:0003918	DNA topoisomerase type II (ATP-hydrolyzing) activity	2	3	0.0134
GO:0003723	RNA binding	19	850	0.0142
GO:0042826	histone deacetylase binding	6	110	0.0142
GO:0019903	protein phosphatase binding	6	123	0.0234
GO:0004849	uridine kinase activity	2	5	0.025
GO:0016879	ligase activity, forming carbon-nitrogen bonds	4	50	0.025
GO:0008327	methyl-CpG binding	3	25	0.0317
GO:0044183	protein folding chaperone	3	25	0.0317
GO:0042802	identical protein binding	30	1754	0.034
GO:0008494	translation activator activity	2	7	0.0384
GO:0003729	mRNA binding	7	198	0.049
**Cellular component**				
GO:0044428	nuclear part	117	4359	2.28E-26
GO:0031981	nuclear lumen	109	4030	3.48E-24
GO:0070013	intracellular organelle lumen	123	5162	5.11E-24
GO:0005654	nucleoplasm	97	3446	4.01E-22
GO:0005634	nucleus	138	6892	1.69E-21

GO:0043226	organelle	181	12432	6.80E-19
GO:0044422	organelle part	155	9111	8.73E-19
GO:0044446	intracellular organelle part	152	8882	2.97E-18
GO:0044424	intracellular part	189	13996	6.57E-18
GO:0043229	intracellular organelle	177	12193	3.35E-17
GO:0005829	cytosol	107	4958	1.75E-16
GO:0043232	intracellular non-membrane-bounded organelle	94	4005	4.54E-16
GO:0043231	intracellular membrane-bounded organelle	160	10365	1.83E-15
GO:0043227	membrane-bounded organelle	164	11244	1.37E-13
GO:0032991	protein-containing complex	97	4792	1.43E-12
GO:1990904	ribonucleoprotein complex	35	770	3.53E-12
GO:0044464	cell part	193	16244	4.77E-11
GO:0005730	nucleolus	33	926	8.32E-09
GO:0005737	cytoplasm	149	11238	1.05E-06
GO:0044444	cytoplasmic part	131	9377	1.69E-06
GO:0030684	preribosome	9	74	2.66E-06
GO:0005694	chromosome	28	950	8.16E-06
GO:0030687	preribosome, large subunit precursor	6	24	8.16E-06
GO:0016607	nuclear speck	17	381	8.88E-06
GO:0044452	nucleolar part	12	180	8.88E-06
GO:0016604	nuclear body	24	742	1.08E-05
GO:0005635	nuclear envelope	18	446	1.48E-05
GO:0015630	microtubule cytoskeleton	30	1118	1.70E-05
GO:0044451	nucleoplasm part	29	1073	2.22E-05
GO:0031965	nuclear membrane	14	300	4.72E-05
GO:0005856	cytoskeleton	42	2068	0.00011
GO:1904813	ficolin-1-rich granule lumen	9	125	0.00011
GO:0044427	chromosomal part	23	819	0.00015
GO:0044430	cytoskeletal part	34	1547	0.00019
GO:0000785	chromatin	16	489	0.00059
GO:0071682	endocytic vesicle lumen	4	19	0.0009
GO:0001650	fibrillar center	8	136	0.0012
GO:0005681	spliceosomal complex	9	187	0.0018
GO:0060205	cytoplasmic vesicle lumen	12	340	0.0025
GO:0031967	organelle envelope	25	1146	0.0027
GO:0015030	Cajal body	5	57	0.0039
GO:0000793	condensed chromosome	9	215	0.0042
GO:0016581	NuRD complex	3	14	0.0059
GO:0005874	microtubule	12	385	0.0061
GO:0071013	catalytic step 2 spliceosome	6	99	0.0061
GO:0048471	perinuclear region of cytoplasm	16	670	0.0129
GO:0070545	PeBoW complex	2	4	0.0131
GO:0099512	supramolecular fiber	19	873	0.0131
GO:0012505	endomembrane system	62	4347	0.0138
GO:0030496	midbody	7	165	0.0141
GO:0005819	spindle	10	322	0.0151
GO:0098687	chromosomal region	9	272	0.0165
GO:0072686	mitotic spindle	5	87	0.0179
GO:0000228	nuclear chromosome	13	514	0.0189
GO:0000127	transcription factor TFIIIC complex	2	6	0.0205
GO:0044295	axonal growth cone	3	26	0.0222
GO:0044454	nuclear chromosome part	12	480	0.0283
GO:0005815	microtubule organizing center	15	683	0.0306
GO:0030686	90S preribosome	3	30	0.0306
GO:0042555	MCM complex	2	8	0.0306
GO:0042641	actomyosin	4	63	0.0311
GO:0031256	leading edge membrane	6	150	0.0325
GO:0031252	cell leading edge	10	371	0.0332

GO:1990391	DNA repair complex	3	32	0.0336
GO:0005844	polysome	4	66	0.034
GO:0035770	ribonucleoprotein granule	7	204	0.034
GO:0000346	transcription export complex	2	10	0.0388
GO:0034774	secretory granule lumen	9	323	0.0388
GO:0044449	contractile fiber part	7	212	0.0396
GO:0035861	site of double-strand break	3	36	0.0417
GO:0001726	ruffle	6	164	0.0422
GO:0005643	nuclear pore	4	72	0.0422
GO:0030016	myofibril	7	216	0.0422
GO:0030688	preribosome, small subunit precursor	2	11	0.0422
GO:0044297	cell body	12	526	0.0444
GO:0005813	centrosome	11	468	0.049
**Reactome Pathway**				
HSA-1640170	Cell Cycle	22	586	0.00012
HSA-8953854	Metabolism of RNA	21	652	0.0013
HSA-3371556	Cellular response to heat stress	7	85	0.0081
HSA-69278	Cell Cycle, Mitotic	16	483	0.0081
HSA-72203	Processing of Capped Intron-Containing Pre-mRNA	11	234	0.0081
HSA-3000484	Scavenging by Class F Receptors	3	6	0.009
HSA-168254	Influenza Infection	8	148	0.0167
HSA-68886	M Phase	12	343	0.0189
HSA-73894	DNA Repair	11	290	0.0189
HSA-3700989	Transcriptional Regulation by TP53	12	359	0.0241
HSA-3371511	HSF1 activation	3	12	0.0253
HSA-68884	Mitotic Telophase/Cytokinesis	3	14	0.0319
HSA-72163	mRNA Splicing - Major Pathway	8	178	0.0319
HSA-159236	Transport of Mature mRNA derived from an Intron-Containing Transcript	5	69	0.0333
HSA-1643685	Disease	22	1018	0.0333
HSA-168253	Host Interactions with Influenza Factors	4	39	0.0333
HSA-168276	NS1 Mediated Effects on Host Pathways	4	37	0.0333
HSA-2173782	Binding and Uptake of Ligands by Scavenger Receptors	4	40	0.0333
HSA-4615885	SUMOylation of DNA replication proteins	4	40	0.0333
HSA-5653656	Vesicle-mediated transport	16	649	0.0333
HSA-68877	Mitotic Prometaphase	8	190	0.0333
HSA-69481	G2/M Checkpoints	7	148	0.0333
HSA-72172	mRNA Splicing	8	186	0.0333
HSA-72312	rRNA processing	8	199	0.0333
HSA-8868773	rRNA processing in the nucleus and cytosol	8	189	0.0333
HSA-8953897	Cellular responses to external stimuli	13	459	0.0333
HSA-445144	Signal transduction by L1	3	20	0.034
HSA-5633007	Regulation of TP53 Activity	7	159	0.034
HSA-5663205	Infectious disease	11	363	0.034
HSA-72202	Transport of Mature Transcript to Cytoplasm	5	77	0.034
HSA-74160	Gene expression (Transcription)	26	1366	0.034
HSA-2262752	Cellular responses to stress	11	384	0.0441
HSA-8956321	Nucleotide salvage	3	23	0.0445
HSA-5693571	Nonhomologous End-Joining (NHEJ)	4	52	0.0472

Table S2. KEGG pathway enrichment analysis of motif-centric phosphorylated sites with multiple kinases using DAVID (related to Figure 6a).

Pathway	FDR	Count
RNA transport	8.87E-22	100
Spliceosome	4.60E-20	82
Biosynthesis of antibiotics	1.09E-11	96
Ribosome	1.82E-10	68
Carbon metabolism	5.04E-10	59
Protein processing in endoplasmic reticulum	3.91E-09	76
Ribosome biogenesis in eukaryotes	5.58E-08	46
Proteasome	1.37E-07	29
Biosynthesis of amino acids	3.97E-07	39
mRNA surveillance pathway	8.48E-07	45
DNA replication	1.31E-05	23
RNA degradation	3.46E-05	37
Cell cycle	8.17E-05	51
Alzheimer's disease	1.80E-04	63
Pyruvate metabolism	4.68E-04	22
Cysteine and methionine metabolism	6.49E-04	21
Mismatch repair	0.0011	15
Glycolysis / Gluconeogenesis	0.0014	30
Nucleotide excision repair	0.0022	23
Citrate cycle (TCA cycle)	0.0025	17
Oocyte meiosis	0.0036	42
Ubiquitin mediated proteolysis	0.0047	49
Huntington's disease	0.0049	64
Aminoacyl-tRNA biosynthesis	0.0053	28
Parkinson's disease	0.0053	50
Metabolic pathways	0.0074	316
ErbB signaling pathway	0.0135	33
Insulin signaling pathway	0.0154	47
Pentose phosphate pathway	0.0154	15
Viral carcinogenesis	0.0358	63
Oxidative phosphorylation	0.0358	44
Pathogenic Escherichia coli infection	0.0358	21
Epstein-Barr virus infection	0.0358	41
Fatty acid metabolism	0.0370	20
Endocytosis	0.0370	72

Table S3. The information on the peptide amount in each TMT channel for all experiments (related to Star Methods).

Figure	Experiment	TMT channel	Peptide amount	Cell condition	Kinase reaction	Raw files
Figure 2	Exp. 1	126	25 µg	DMSO	N.A.	170705Fe_DCKi_CK2_TMT6_1_MS3.raw
		127	25 µg	CKi-treated	N.A.	170705Fe_DCKi_CK2_TMT6_2_MS3.raw
		128	25 µg	Motif-centric	CK2	
		129	25 µg	DMSO	N.A.	
		130	25 µg	CKi-treated	N.A.	
		131	25 µg	Motif-centric	CK2	
Figure S2	Exp. 2	126	25 µg	DMSO	N.A.	170715Fe_F50_PKA_TMT6_1.raw
		127	25 µg	forskolin -treated	N.A.	170715Fe_F50_PKA_TMT6_2.raw
		128	25 µg	Motif-centric	PKA	170715Fe_F50_PKA_TMT6_3.raw
		129	25 µg	DMSO	N.A.	
		130	25 µg	forskolin -treated	N.A.	
		131	25 µg	Motif-centric	PKA	
Figure S3b	Exp. 3	126		Empty		170620Fe_3plex_noPV_MS2.raw
		127	25 µg	EGF-treated	N.A.	
		128	25 µg	EGF/Afatinib-treated	N.A.	
		129		Empty		
		130		Empty		
		131		Empty		
	Exp. 4	126		Empty		170620Fe_3plex_PV50_MS2.raw
		127	25 µg	EGF-treated	N.A.	
		128	25 µg	EGF/Afatinib-treated	N.A.	
		129		Empty		
		130	50 µg	PV-treated	N.A.	
		131		Empty		
	Exp. 5	126		Empty		170620Fe_3plex_PV100_MS2.raw
		127	25 µg	EGF-treated	N.A.	
		128	25 µg	EGF/Afatinib-treated	N.A.	
		129		Empty		
		130	100 µg	PV-treated	N.A.	
		131		Empty		
	Exp. 6	126		Empty		170620Fe_3plex_PV150_MS2.raw
		127	25 µg	EGF-treated	N.A.	
		128	25 µg	EGF/Afatinib-treated	N.A.	
		129		Empty		
		130	150 µg	PV-treated	N.A.	
		131		Empty		
Figure 3,	Exp. 7	126	25 µg	EGF-treated	N.A.	170915Fe_EGFAFA_PV200_MS3_F1.raw

Figure S3a, S3c		127	25 µg	EGF/Afatinib-treated	N.A.	170915Fe_EGFAFA_PV200_MS3_F2.raw
		128	25 µg	EGF-treated	N.A.	170915Fe_EGFAFA_PV200_MS3_F3.raw
		129	25 µg	EGF/Afatinib-treated	N.A.	170915Fe_EGFAFA_PV200_MS3_F4.raw
		130		Empty		170915Fe_EGFAFA_PV200_MS3_F5.raw
		131	25 µg	PV-treated	N.A.	
	Exp. 8	126	25 µg	EGF-treated	N.A.	170915Fe_EGFAFA_EGFR_MS3_F1.raw
		127	25 µg	EGF/Afatinib-treated	N.A.	170915Fe_EGFAFA_EGFR_MS3_F2.raw
		128	25 µg	EGF-treated	N.A.	170915Fe_EGFAFA_EGFR_MS3_F3.raw
		129	25 µg	EGF/Afatinib-treated	N.A.	170915Fe_EGFAFA_EGFR_MS3_F4.raw
		130		Empty		170915Fe_EGFAFA_EGFR_MS3_F5.raw
		131	25 µg	Motif-centric	EGFR	
	Exp. 9	126	25 µg	EGF-treated	N.A.	170915Fe_EGFAFA_SRC_MS3_F1.raw
		127	25 µg	EGF/Afatinib-treated	N.A.	170915Fe_EGFAFA_SRC_MS3_F2.raw
		128	25 µg	EGF-treated	N.A.	170915Fe_EGFAFA_SRC_MS3_F3.raw
		129	25 µg	EGF/Afatinib-treated	N.A.	170915Fe_EGFAFA_SRC_MS3_F4.raw
		130		Empty		170915Fe_EGFAFA_SRC_MS3_F5.raw
		131	25 µg	Motif-centric	SRC	
Figure 5, Figure S4a	Exp. 10	126	25 µg	EGF-treated	N.A.	170921Fe_EAfa_CK2_TMT6_1.raw
		127	25 µg	EGF/Afatinib-treated	N.A.	170921Fe_EAfa_CK2_TMT6_2.raw
		128	25 µg	Motif-centric	CK2	170921Fe_EAfa_CK2_TMT6_3.raw
		129	25 µg	EGF-treated	N.A.	
		130	25 µg	EGF/Afatinib-treated	N.A.	
		131	25 µg	Motif-centric	CK2	
	Exp. 11	126	25 µg	EGF-treated	N.A.	170921Fe_EAfa_CDK_TMT6_1.raw
		127	25 ug	EGF/Afatinib-treated	N.A.	170921Fe_EAfa_CDK_TMT6_3.raw
		128	25 ug	Motif-centric	CDK1	
		129	25 ug	EGF-treated	N.A.	
		130	25 ug	EGF/Afatinib-treated	N.A.	
		131	25 ug	Motif-centric	CDK1	
	Exp. 12	126	25 ug	EGF-treated	N.A.	170921Fe_EAfa_ERK_TMT6_1.raw
		127	25 ug	EGF/Afatinib-treated	N.A.	170921Fe_EAfa_ERK_TMT6_2.raw
		128	25 ug	Motif-centric	ERK2	
		129	25 ug	EGF-treated	N.A.	
		130	25 ug	EGF/Afatinib-treated	N.A.	
		131	25 ug	Motif-centric	ERK2	
	Exp. 13	126	25 ug	EGF-treated	N.A.	170921Fe_EAfa_JNK_TMT6_1.raw
		127	25 ug	EGF/Afatinib-treated	N.A.	170921Fe_EAfa_JNK_TMT6_2.raw
		128	25 ug	Motif-centric	JNK1	
		129	25 ug	EGF-treated	N.A.	
		130	25 ug	EGF/Afatinib-treated	N.A.	
		131	25 ug	Motif-centric	JNK1	
	Exp. 14	126	25 ug	EGF-treated	N.A.	170921Fe_EAfa_p38a_TMT6_1.raw
		127	25 ug	EGF/Afatinib-treated	N.A.	170921Fe_EAfa_p38a_TMT6_3.raw
		128	25 ug	Motif-centric	p38a	

		129	25 ug	EGF-treated	N.A.	
		130	25 ug	EGF/Afatinib-treated	N.A.	
		131	25 ug	Motif-centric	p38a	
	Exp. 15	126	25 ug	EGF-treated	N.A.	170921Fe_EAfa_EGFR_TMT6_1.raw
		127	25 ug	EGF/Afatinib-treated	N.A.	70921Fe_EAfa_EGFR_TMT6_2.raw
		128	25 ug	Motif-centric	EGFR	170921Fe_EAfa_EGFR_TMT6_3.raw
		129	25 ug	EGF-treated	N.A.	
		130	25 ug	EGF/Afatinib-treated	N.A.	
		131	25 ug	Motif-centric	EGFR	
	Exp. 16	126	25 ug	EGF-treated	N.A.	170921Fe_EAfa_SRC_TMT6_1.raw
		127	25 ug	EGF/Afatinib-treated	N.A.	170921Fe_EAfa_SRC_TMT6_2.raw
		128	25 ug	Motif-centric	SRC	170921Fe_EAfa_SRC_TMT6_3.raw
		129	25 ug	EGF-treated	N.A.	
		130	25 ug	EGF/Afatinib-treated	N.A.	
		131	25 ug	Motif-centric	SRC	
Figure 4	Exp. 17	126	5 ug	Un-treated	N.A.	210825ko02Motif_EGFR_Set1_1.raw
		127	10 ug	Un-treated	N.A.	210825ko02Motif_EGFR_Set1_2.raw
		128	25 ug	Un-treated	N.A.	210825ko02Motif_EGFR_Set1_3.raw
		129	60 ug	Un-treated	N.A.	
		130		Empty		
		131	5 ug	Motif-centric	EGFR	
	Exp. 18	126	5 ug	Un-treated	N.A.	210825ko02Motif_EGFR_Set2_1.raw
		127	10 ug	Un-treated	N.A.	210825ko02Motif_EGFR_Set2_2.raw
		128	25 ug	Un-treated	N.A.	210825ko02Motif_EGFR_Set2_3.raw
		129	60 ug	Un-treated	N.A.	
		130		Empty		
		131	25 ug	Motif-centric	EGFR	
	Exp. 19	126	5 ug	Un-treated	N.A.	210825ko02Motif_EGFR_Set3_1.raw
		127	10 ug	Un-treated	N.A.	210825ko02Motif_EGFR_Set3_2.raw
		128	25 ug	Un-treated	N.A.	210825ko02Motif_EGFR_Set3_3.raw
		129	60 ug	Un-treated	N.A.	
		130		Empty		
		131	125 ug	Motif-centric	EGFR	
